# Engineered Macrophage Membrane-Coated Nanoparticles for Hepatic Ischemia–Reperfusion Injury Therapeutics

**DOI:** 10.34133/bmr.0212

**Published:** 2025-05-23

**Authors:** Long Yang, Weiwei Li, Zhen Huang, Yinping Zhao, Zhenwen Sun, Haoyu Wang, Longpo Cao, Jiao Lu, Ruirui Sun, Xiang Ma, Tianxin Shao, Xixi Wu, Siqi He, Zuojin Liu

**Affiliations:** ^1^ Hepatobiliary Surgery, The Second Affiliated Hospital of Chongqing Medical University, Chongqing, China.; ^2^ College of Biomedical Engineering, Chongqing Medical University, Chongqing, China.; ^3^ Department of Orthopedic Oncology, Shanghai General Hospital, School of Medicine, Shanghai Jiao Tong University, Shanghai, China.

## Abstract

Hepatic ischemia–reperfusion injury (HIRI) is a common perioperative complication occurring after liver transplantation and can lead to further problems such as early allograft dysfunction (EAD). Currently, the treatment options for HIRI are extremely limited. In this study, we used bioinformatics analysis to elucidate the critical role of neutrophil chemokines (CXC chemokines) in HIRI. By analyzing sequencing data from the hepatic tissue of posttransplant patients with EAD and the reperfused animal model, we discovered that hepatocytes and macrophages are the primary cells secreting CXC chemokines, and the activation of the nuclear factor kappa B (NF-κB) signaling pathway is the main driver of their secretion. Melatonin (MT) can protect cells from oxidative harm while also inhibiting NF-κB signaling, suggesting its potential to ameliorate HIRI. Accordingly, we designed a nanoparticle platform coated with genetically engineered macrophage membranes—called CXCR2-MM@PLGA/MT—to target the cells secreting CXC chemokines. CXCR2 overexpression on the macrophage membranes not only enhanced the targeting capacity of the nanoparticles but also prevented neutrophil infiltration via the scavenging of CXC chemokines. Meanwhile, the MT delivered to the site of injury successfully attenuated CXC chemokine release after macrophage polarization and hepatocyte necrosis by inhibiting NF-κB phosphorylation and inducing antioxidant effects. Through the synergistic effects of MT and the CXCL/CXCR axis-blocking function of the engineered nanoparticles, CXCR2-MM@PLGA/MT attenuated the aggregation of neutrophils at the site of injury, markedly reducing local inflammation and cellular damage following HIRI. This engineered cellular nanoparticle-based therapy could thus serve as a safe, effective, and cost-efficient strategy for treating HIRI.

## Introduction

Liver transplantation (LT) has become the standard treatment for patients with end-stage liver disease, and technological advancements in recent decades have led to improved patient outcomes after this procedure [1]. However, early allograft dysfunction (EAD) remains a major complication of LT, negatively affecting both patient and graft survival [2]. EAD is a multifactorial condition influenced by donor risk factors, surgical techniques, and the severity of end-stage liver disease, and its severity is often evaluated using predictive scoring models [3]. One of the key contributors to the pathogenesis of EAD is hepatic ischemia–reperfusion injury (HIRI), a complex pathological process that amplifies immune responses and potentially leads to both acute and chronic rejection [4]. Notably, HIRI is responsible for up to 10% of all early liver transplant failures [5]. A critical factor in HIRI development is the inflammatory burst, which triggers a strong inflammatory response [6]. This leads to the uncontrolled release of pro-inflammatory cytokines, resulting in the excessive activation of immune cells, hepatocyte death, and severe tissue damage [7]. Given the central role of pro-inflammatory cytokines in HIRI pathogenesis, researchers have devoted considerable effort toward targeting these cytokines for therapeutic purposes [8]. In this context, small-molecule cytokine inhibitors and neutralizing antibodies have shown immense therapeutic potential [9,10]. However, implementing the comprehensive blockade of multiple cytokines is often impractical [11]. As a result, the targeted inhibition of key pro-inflammatory cytokines and signaling pathways involved in the development and pathogenesis of HIRI has also emerged as a key issue [12]. Therefore, novel strategies that address these challenges are urgently needed for effective HIRI treatment.

Melatonin (MT), a hormone produced by the pineal gland, is recognized for its potent free radical scavenging and antioxidant properties [13]. Notably, MT has been shown to inhibit the nuclear factor kappa B (NF-κB) signaling pathway, which attenuates HIRI in rat models, highlighting its potential as a therapeutic agent to mitigate the inflammatory cascade associated with HIRI [14]. However, the short half-life of MT in the bloodstream limits its therapeutic efficacy [15]. To overcome this hurdle, researchers often administer high doses of MT (20 mg·kg^−1^) intravenously, which increases the risk of side effects such as rhythm disorders [16]. Overall, the poor solubility of MT and its short circulation time and adverse effects have hindered its clinical success as a pharmacological intervention. Poly(lactic-co-glycolic acid) (PLGA), a copolymer of glycolic acid and lactic acid, has been approved by the U.S. Food and Drug Administration for clinical use [17]. In fact, PLGA has been identified as an ideal carrier for MT due to its biosafety [18] and biocompatibility [19]. In recent years, natural cell membrane-coated nanoparticles (NPs) have emerged as a new class of biomimetic particles for coating synthetic NPs [20,21]. These biomimetic particles retain some functional properties of their source cell membranes, improving the circulation stability [22], immune evasion capacity [23], and biocompatibility of NPs [24]. Several types of cell membrane-coated NPs have been developed to target inflammatory mediators, suppress inflammatory responses, or regulate inflammatory pathways to treat HIRI [25,26]. Among them, macrophage membrane-coated NPs are particularly promising due to their ability to scavenge and neutralize pro-inflammatory cytokines owing to the presence of inflammatory factor receptors on the macrophage membrane surface [27]. Genetically engineered cell membrane-coated NPs offer additional benefits over conventional synthetic nanomaterials loaded with antibodies and small-molecule drugs, such as improved biocompatibility and the ability to express proteins tailored to specific therapeutic needs [28].

**Fig. 1.**
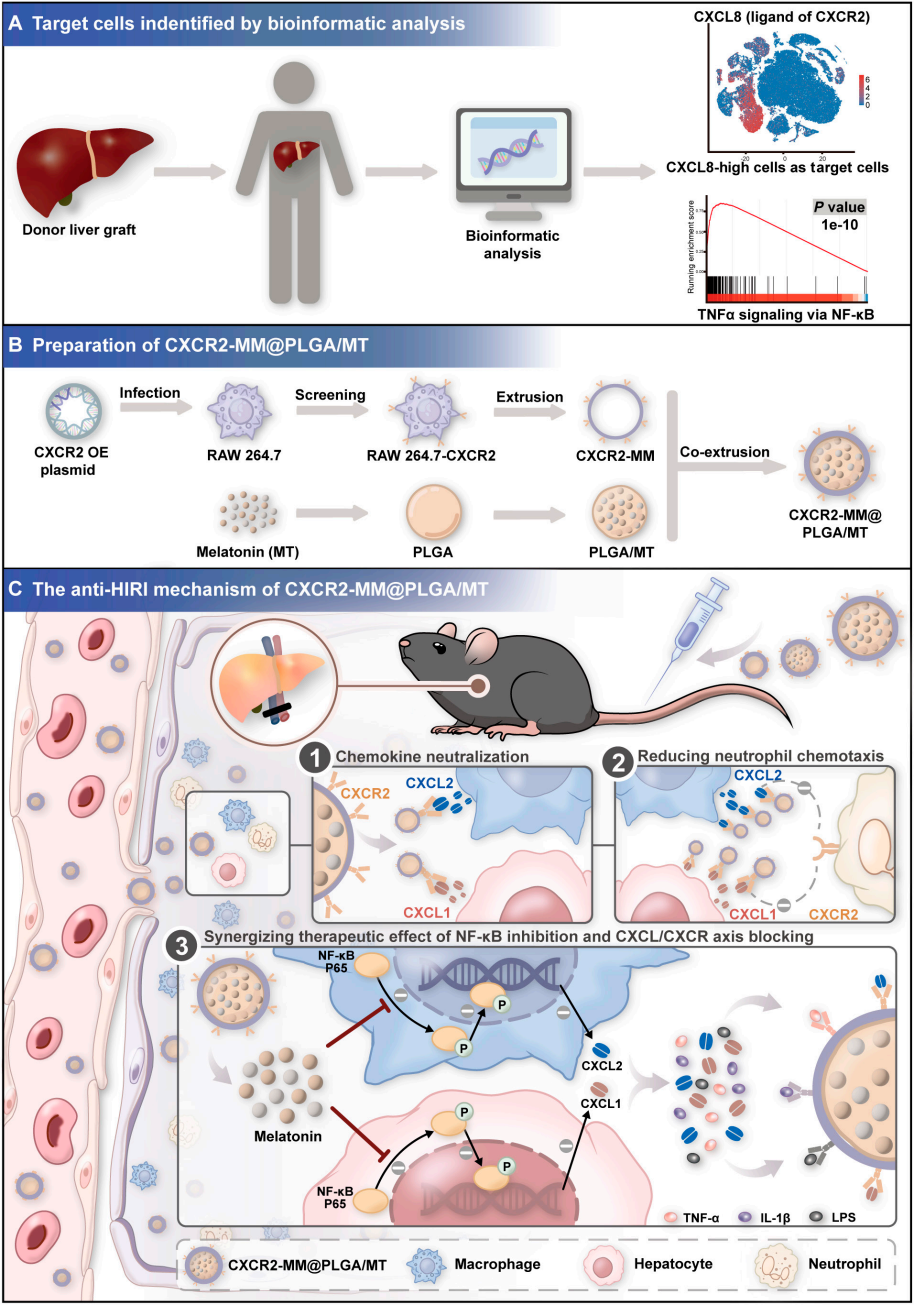
Schematic representation of the mechanism of CXCR2-MM@PLGA/MT in the HIRI. This strategy offers several key advantages: (A) Overexpression of CXCR2 on macrophage membranes enhances the ability of the nanoparticles to target the site of inflammation. (B) CXCR2 overexpression on macrophage membranes competitively binds to CXC chemokines released by hepatocytes and macrophages, effectively blocking the CXCL–CXCR signaling axis and inhibiting neutrophil infiltration. (C) The release of the therapeutic agent inhibits the NF-κB pathway, reducing the expression of CXC chemokines at the source. Simultaneously, the macrophage membrane acts as a nanodecoy, further mitigating the inflammatory cascade by sequestering additional cytokines. Together, the combination of therapeutic agents and engineered macrophage membranes synergistically reduces inflammation and necrosis following HIRI.

In this study, we explored the critical role of neutrophil chemokines (CXC chemokines) in HIRI through bioinformatics analysis. By analyzing sequencing data from the hepatic tissues of posttransplant patients with EAD and reperfused mice, we identified hepatocytes and macrophages as the primary sources of CXC chemokine secretion, with the activation of the NF-κB signaling pathway being the main driver of this process. Building on these insights, we developed an NP platform coated with genetically engineered macrophage membranes—called CXCR2-MM@PLGA/MT—to specifically target the key cells secreting CXC chemokines. The macrophage cell membranes conferred this platform with biomimetic properties, improving the circulatory stability of the NPs and enabling their targeted delivery to inflammatory sites. The overexpression of CXCR2 on the macrophage membranes not only enhanced the targeting capacity of NPs to the sites of injury but also reduced neutrophil infiltration through the sequestration of CXC chemokines. Meanwhile, the MT delivered by the NPs attenuated CXC chemokine release following macrophage polarization and hepatocyte necrosis by inhibiting NF-κB signaling and generating antioxidant effects. The CXCR2-MM@PLGA/MT NPs not only retained the functional properties of previous macrophage membrane-coated NPs, such as the scavenging of pro-inflammatory cytokines and immune evasion, but also showed an enhanced ability to target and neutralize the key inflammatory factors (CXC chemokines) involved in HIRI. This approach improved drug stability, enhanced targeting, and increased the accumulation of the drug at the site of inflammation, thus reducing side effects. This engineered cellular NP-based therapy could thus serve as a safe, effective, and cost-efficient strategy for treating HIRI (Fig. 1).

## Materials and Methods

### Lentiviral production

According to a previous study [29], briefly, human embryonic kidney (HEK) 293T cells (American Type Culture Collection [ATCC]) were inoculated into T75 cell culture flasks at 50% fusion 12 h prior to transfection. The transfection plasmid pCDH-CMV-CXCR2-EF1-Puro (LeapWal) was mixed with the packaging plasmid (pMDL:pRev:pMD2.G = 1:1:1 weight ratio) in 5 ml of microcosmic medium at a 1.5:1 weight ratio (concentration of the transfected plasmid = 4 μg/ml). The plasmids were cotransfected into HEK293T cells using Lipofectamine 2000 (Thermo Fisher Scientific). Cells were cultured in Dulbecco’s modified Eagle’s medium (DMEM; Gibco) at 37 °C under 5% CO_2_. After 6 h, the medium was changed to DMEM containing 10% fetal bovine serum (FBS, Procell) and 1% penicillin-streptomycin (Beyotime). After transfection for 48 and 72 h, culture supernatants containing virus particles were collected. Cellular debris in the supernatant was centrifuged at 1,000 × *g* for 15 min and then filtered through a 0.45-μm filter (Millipore). After filtration, the virus particles were separated using a virus concentrate (LeapWal) and resuspended in 100 μl of 1× phosphate-buffered saline (PBS). The concentrated virus suspension was stored at −80 °C for subsequent use.

### Generation of macrophages overexpressing CXCR2

RAW 264.7, a mouse monocyte macrophage leukemia cell line, was obtained from ATCC. To initiate lentiviral transfection, RAW 264.7 cells were inoculated into 6-well plates at a density of 1 × 10^5^ cells/well and cultured in DMEM supplemented with 10% fetal bovine serum for 24 h. The cells were then infected with the lentiviral suspension and re-cultured in fresh culture medium after 24 h. Next, the cultured cells were treated with puromycin at an initial concentration of 1 μg/ml to screen for genetically engineered RAW 264.7-CXCR2 cells with high infection efficiency and purity.

### Cell membrane derivation

Membranes of RAW 264.7 cells and RAW 264.7-CXCR2 cells were derived based on a previously reported protocol [30]. Briefly, cells were suspended in hypotonic lysis buffer containing 30 mM Tris-HCl (pH 7.5), 225 mM d-mannitol, 75 mM sucrose, 0.2 mM EGTA (all from Solarbio), and protease and phosphatase inhibitors (Beyotime). The cells were then disrupted using a Dounce homogenizer. The homogenized solution was centrifuged at 10,000 × *g* for 25 min at 4 °C. The precipitate was discarded and the supernatant was washed at 150,000 × *g* for 30 min at 4 °C. After centrifugation, the membrane was resuspended in 37 ml of 0.2 mM ethylenediaminetetraacetic acid (EDTA, Beyotime) solution and centrifuged at 150,000 × *g* for 30 min at 4 °C. The final membrane storage solution was resuspended in 1 ml of 0.2 mM EDTA. The concentration of membrane proteins was determined using a BCA kit (Beyotime). Store the membrane suspension at −80 °C for subsequent use.

### Preparation of cell membrane-coated NPs

PLGA/MT and membrane-coated PLGA/MT NPs were prepared according to a previously reported protocol [30]. Briefly, PLGA (50 mg/ml, 1 ml, Daigangbio) in dichloromethane with MT (2 mg/ml, 1 ml, MCE) was added to 5% w/v PVA (RHAWN) solution by sonication (42 kHz, 100 W) for 5 min in ice bath conditions to obtain an emulsion. The mixture was then poured into 4 ml of 1% w/v PVA solution and stirred in the open air until the dichloromethane evaporated completely. The solutions were prepared and dialyzed in ultrapure water using a dialysis bag (3.5 kDa molecular weight cutoff [MWCO], Millipore) for 12 h to remove free MT and organic solvents. PLGA/MT NPs were collected after centrifugation at 15,000 × *g* for 15 min and freeze-dried to obtain pure NPs. To prepare membrane-coated PLGA/MT NPs, the macrophage membranes were mixed with PLGA NPs at a membrane protein:PLGA weight ratio of 1:1. The mixtures were then sonicated (42 kHz, 100 W) for 2 min using a bath sonicator (Thermo Fisher Scientific) and finally the mixtures were extruded 15 times through a 200-nm polycarbonate porous membrane using a micro-extruder. After obtaining the membrane-coated PLGA/MT NPs solution by extrusion, the NPs were concentrated to a protein concentration of 10 mg/ml by centrifugation at 4,500 × *g* for 15 min using an Amicon ultracentrifugal filter (30 kDa MWCO, Millipore). The NPs were collected after centrifugation at 15,000 × *g* for 15 min and freeze-dried to obtain pure NPs. Membrane-coated PLGA and PLGA NPs were prepared following the same procedure without the addition of MT. Membrane-coated PLGA/coumarin6 and PLGA/coumarin6 NPs were prepared following the same procedure, coumarin6 was added replacing MT, and the dye concentration was 0.1 wt% of PLGA polymer. Membrane-coated PLGA/DiR and PLGA/Dir NPs were prepared following the same procedure. DiR was added to replace MT, and the dye concentration was 0.1 wt% of PLGA polymer.

### Antibodies

Complete information on all antibodies used in this study is provided in Table S1.

More experimental section information can be found in the Supplementary Materials.

## Results and Discussion

### MT modulates the NF-κB pathway in hepatocytes and macrophages to reduce CXC chemokine expression

**Fig. 2.**
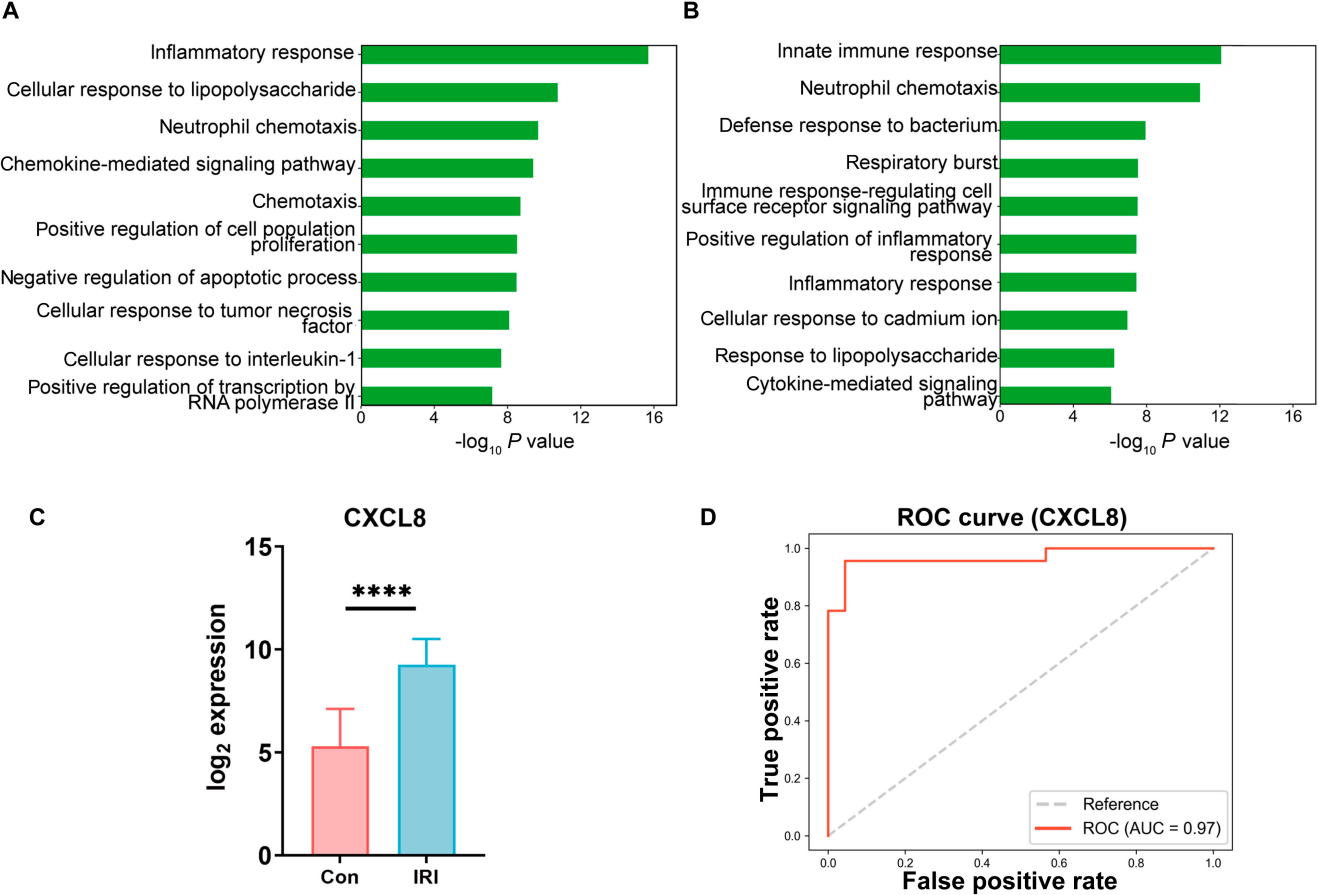
The importance of CXC chemokines in the progression of HIRI. (A) Gene Ontology (GO) enrichment analysis of biological processes for differentially expressed genes in liver tissue following reperfusion compared to pre-reperfusion tissue in the context of HIRI, based on data from the GSE151648 dataset. (B) GO enrichment analysis of biological processes for differentially expressed genes in perfused early allograft dysfunction (EAD) compared to perfused non-EAD tissues, using data from the GSE189539 dataset. (C) CXCL8 gene expression levels in liver tissue following reperfusion compared to pre-reperfusion liver tissue (****P < 0.0001), based on data from the GSE151648 dataset. (D) Receiver operating characteristic (ROC) curve for CXCL8 in the GSE151648 dataset.

To explore the relationship between CXC chemokines and ischemia–reperfusion injury (IRI) following LT, we conducted a GO enrichment analysis of the GSE151648 and GSE189539 datasets. The analysis revealed an up-regulation of neutrophil chemokine activity in posttransplant versus pretransplant hepatic tissues (Fig. 2A). Additionally, a comparison of posttransplant tissues from patients with and without EAD further confirmed the enhancement of neutrophil chemokine activity under EAD (Fig. 2B). These findings suggested that neutrophil chemokines not only are involved in the inflammatory response following LT but also contribute markedly to the critical complications that impact graft survival. CXCL8, also known as interleukin-8 (IL-8), is one of the most extensively studied neutrophil chemokines [31]. There is considerable evidence supporting the role of CXCL8 in attracting neutrophils, primarily through its interaction with the chemokine receptor CXCR [32]. To investigate the potential involvement of CXCL8 in IRI following LT, we performed differential expression analysis and identified the marked up-regulation of CXCL8 in posttransplant versus pretransplant hepatic tissues (Fig. 2C). Subsequently, we examined the diagnostic potential of CXCL8 in LT-related IRI and associated complications by generating receiver operating characteristic (ROC) curves based on the GSE151648 dataset. ROC analysis revealed that CXCL8 exhibited strong diagnostic performance in differentiating IRI samples from controls, highlighting its potential as a diagnostic biomarker for IRI after LT (Fig. 2D). Collectively, these findings demonstrated the marked relationship of CXC chemokines, especially CXCL8, with HIRI and other key posttransplant complications that influence graft survival.

**Fig. 3.**
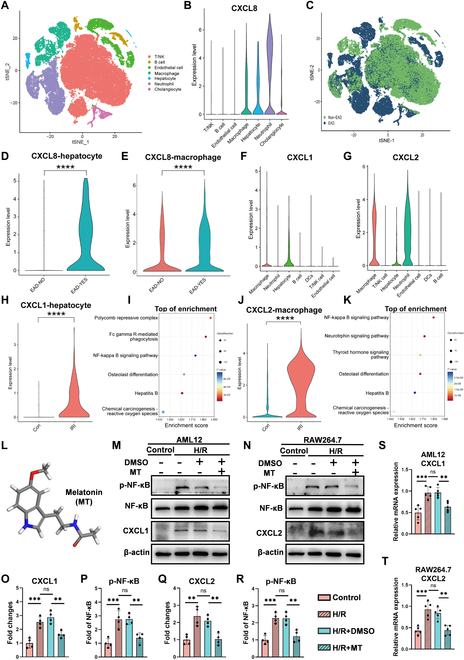
Melatonin modulates the NF-κB pathway in AML12 and RAW 264.7 cells to reduce CXC chemokine expression. (A and B) CXCL8 expression distribution across all cell types and corresponding quantitative analysis results. (C to E) Distribution of CXCL8 expression in early allograft dysfunction (EAD) and non-EAD, with quantitative analysis results (****P < 0.0001). (F and G) Distribution and quantitative analysis of CXCL1 and CXCL2 expression across all cell types. (H and I) CXCL1 expression distribution in hepatocytes from IRI and control groups, with KEGG enrichment analysis of differentially expressed genes (****P < 0.0001). (J and K) CXCL2 expression distribution in macrophages from IRI and control groups, with KEGG enrichment analysis of differentially expressed genes (****P < 0.0001). (L) Three-dimensional structure of melatonin. (M to R) Western blot analysis of p-NF-κB, NF-κB, CXCL1, and CXCL2 expression in AML12 and RAW 264.7 cells treated with hypoxia–reoxygenation (H/R: 3 h in hypoxia and 6 h in reoxygenation), showing their relative expression, with β-actin as a control (“ns” denotes no significance, **P < 0.01, ***P < 0.001). (S and T) Relative mRNA expression levels of CXCL1 and CXCL2 measured by RT-qPCR in AML12 and RAW 264.7 cells treated with hypoxia–reoxygenation (“ns” denotes no significance, **P < 0.01, ***P < 0.001).

To identify the major cell types responsible for CXCL8 secretion in HIRI, we analyzed a single-cell dataset from liver transplant specimens of patients with and without EAD. Single-cell assays revealed that CXCL8 was highly expressed in neutrophils, hepatocytes, and macrophages (Fig. 3A and B). In addition, we found that CXCR2—the receptor for CXCL8—was predominantly expressed in the neutrophil population (Fig. S1A). While neutrophils can secrete CXCL8 upon inflammatory stimulation, our study focused on chemokine production during the initial phase of reperfusion. Therefore, our subsequent investigations were centered on hepatocytes and macrophages. Cellular clustering analysis in patients with and without EAD (Fig. 3C) indicated that neutrophils, hepatocytes, and macrophages play crucial roles in the development of EAD posttransplantation. Moreover, CXCL8 expression was markedly elevated in hepatocytes and macrophages in the EAD group (Fig. 3D and E). To further explore this phenomenon, we analyzed a single-cell dataset from a murine model of HIRI. In rodents, neutrophil chemotaxis is primarily mediated by CXCL1 and CXCL2, since no CXCL8 protein is present [32]. Our single-cell assays demonstrated that CXCL1 was predominantly expressed in hepatocytes, while CXCL2 was highly expressed in macrophages and neutrophils (Fig. 3F and G). Similarly, we examined the expression of CXCR2, the receptor for CXCL1 and CXCL2, and found that CXCR2 is predominantly expressed in neutrophil populations (Fig. S1B). These results were consistent with our earlier findings from the human LT dataset, suggesting that hepatocytes and macrophages are the key contributors to neutrophil chemoattraction during the ischemia–reperfusion phase. To explore the hepatocyte and macrophage signaling pathways involved in HIRI, we conducted differential analyses in hepatocyte and macrophage populations. Kyoto Encyclopedia of Genes and Genomes (KEGG) analysis of differentially expressed genes revealed that the NF-κB signaling pathway was the primary pathway activated during HIRI in both cell types (Fig. 3H to K). To explore the mechanisms of inflammation in other tissues during HIRI, we analyzed the GSE235367 dataset, which contained information regarding the immune status of lung tissues during HIRI. The results showed that during HIRI, the expression of CXCL1 and CXCL2 remained low in lung tissues and did not differ markedly between the sham-operated and HIRI groups (Fig. S2). This indicated that neutrophil chemotaxis in the liver tissue following HIRI is not affected by inflammation in other tissues.

MT (Fig. 3L), a heterocyclic molecule with potent antioxidant properties, has been shown to inhibit the NF-κB signaling pathway and ameliorate HIRI in rat models [14]. Our previous experiments demonstrated the marked activation of the NF-κB signaling pathway in hepatocytes and macrophages during HIRI. Thus, we selected hepatocytes (AML-12) and macrophages (RAW 264.7) to investigate the cellular mechanisms through which MT modulates NF-κB activation and suppresses CXC chemokine secretion. We created an in vitro ischemia–reperfusion model by exposing AML-12 and RAW 264.7 cells to conditions of hypoxia and reoxygenation before MT treatment. The hypoxia marker hypoxia inducible factor-1 (HIF-1α) confirmed the successful establishment of the in vitro IRI model (Fig. S3). Western blot and real-time quantitative polymerase chain reaction (RT-qPCR) analyses revealed that MT markedly inhibited the activation of the NF-κB pathway. Furthermore, it prevented the up-regulation of CXC chemokine secretion induced by hypoxia–reoxygenation in both AML-12 hepatocytes and RAW 264.7 macrophages (Fig. 3M to T). Subsequently, we examined the changes in macrophage polarization and discovered a marked increase in inducible nitric oxide synthase (iNOS) expression after hypoxia–reoxygenation, which indicated M1-type polarization. However, MT treatment markedly reduced iNOS expression, suggesting that MT attenuates the degree of M1 polarization induced by hypoxia–reoxygenation (Fig. S4).

In summary, the results of our bioinformatics analyses indicated that hepatocytes and macrophages play a central role in neutrophil recruitment and chemoaggregation during HIRI in both human LT-associated EAD and animal models. Macrophages and hepatocytes can secrete CXC chemokines during HIRI to induce neutrophil infiltration. This central role of CXC chemokines in HIRI makes the CXCL–CXCR axis a valuable therapeutic target for HIRI. However, to our knowledge, there are few effective drugs that target this axis for the clinical treatment of HIRI [33,34]. Our results showed that the NF-κB signaling pathway is the main pathway activated in macrophages and hepatocytes during HIRI. Previous studies have also shown that the NF-κB signaling pathway regulates the expression of various cytokines and chemokines, including CXCL1and CXCL2 [35]. In addition, our hypoxia–reoxygenation cellular model, which mimicked IRI in vitro, revealed that MT can effectively inhibit the activation of hepatocytes and macrophages, reducing the secretion of CXC chemokines by regulating the activation of the NF-κB pathway. Based on these findings, we developed a therapeutic strategy for HIRI by targeting the NF-κB pathway.

### Preparation and characterization of CXCR2-MM@PLGA/MT

**Fig. 4.**
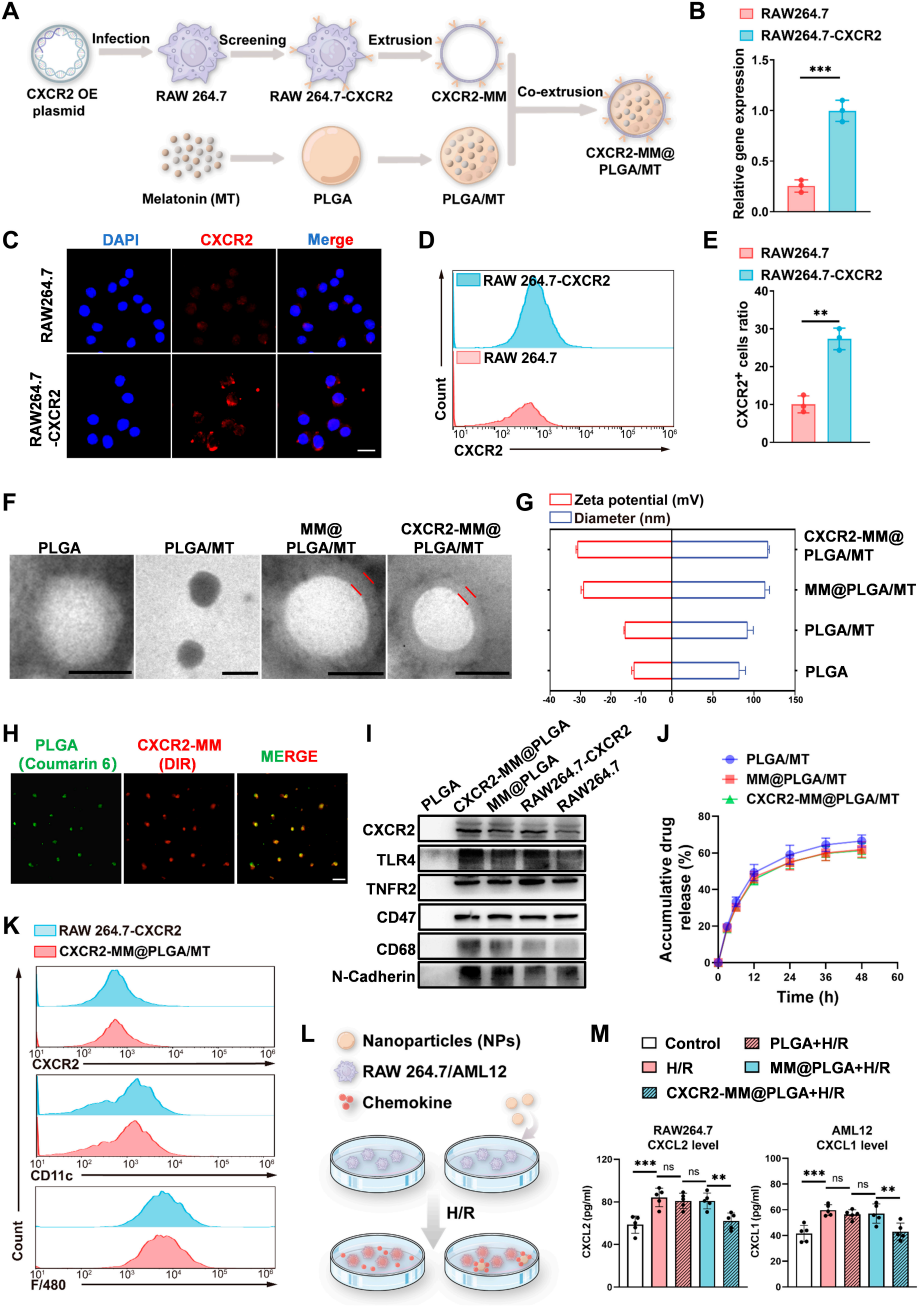
Preparation and characterization of CXCR2-MM@PLGA/MT. (A) Schematic representation of the preparation process for CXCR2-MM@PLGA/MT NPs. (B) RT-qPCR analysis of *CXCR2* mRNA expression in RAW 264.7 and RAW 264.7-CXCR2 cells (***P < 0.001). (C) Immunofluorescence staining for CXCR2 in RAW 264.7 and RAW 264.7-CXCR2 cells. Scale bar = 20 nm. (D and E) Flow cytometry analysis and statistical representation of CXCR2^+^ cell ratios in RAW 264.7 and RAW 264.7-CXCR2 cells (**P < 0.01). (F) Representative transmission electron microscopy (TEM) images of PLGA, PLGA/MT, MM@PLGA/MT, and CXCR2-MM@PLGA/MT NPs. Scale bar = 100 nm. (G) Analysis of average diameter and ζ-potential for CXCR2-MM@PLGA/MT, MM@PLGA/MT, PLGA/MT, and PLGA NPs. (H) Fluorescence colocalization of the RAW 264.7-CXCR2-MM “shell” (red) and PLGA “core” (green). Scale bar = 20 nm. (I) Western blot analysis of CXCR2, TNFR2, TLR4, CD47, and CD68 protein expression in PLGA, MM@PLGA, CXCR2-MM@PLGA NPs, as well as in RAW 264.7 and RAW 264.7-CXCR2 cells, with N-cadherin as a housekeeping marker. (J) Drug release profiles of PLGA/MT, MM@PLGA/MT, and CXCR2-MM@PLGA/MT in vitro. (K) Flow cytometry analysis of CXCR2 and macrophage biomarkers (CD11c, F4/80) in RAW 264.7-CXCR2 cells, CXCR2-MM, and CXCR2-MM@PLGA/MT. (L and M) ELISA analysis of CXCL1 and CXCL2 levels in the supernatants of AML12 and RAW 264.7 cells treated with hypoxia–reoxygenation and subsequent incubation with PLGA, MM@PLGA, and CXCR2-MM@PLGA NPs (“ns” denotes no significance, **P < 0.01, ***P < 0.001).

The process for synthesizing engineered CXCR2-MM@PLGA/MT NPs is depicted in Fig. 4A. To generate the genetically engineered macrophage membranes, we first produced CXCR2-overexpressing macrophages (RAW 264.7-CXCR2) through the lentiviral transduction of a vector encoding the *CXCR2* gene. Successfully transfected cells were selected using puromycin. To confirm the efficiency of CXCR2 transfection, we performed RT-qPCR, which demonstrated a marked increase in *CXCR2* mRNA expression in RAW 264.7-CXCR2 cells (Fig. 4B). Immunofluorescence analysis further confirmed the localization of CXCR2 on macrophage membranes, with markedly increased CXCR2 fluorescence signals observed in RAW 264.7-CXCR2 cells versus controls (Fig. 4C). This demonstrated the successful up-regulation of CXCR2 expression in transfected cells. These results were validated by flow cytometry analysis, which showed that CXCR2^+^ cells accounted for 27.34% of all RAW 264.7-CXCR2 macrophages, representing a 2.72-fold increase compared to the control RAW 264.7 cell population (Fig. 4D and E). Simultaneously, PLGA NPs containing MT (PLGA/MT) were synthesized using the nanoprecipitation method [36]. Using UV/Vis (ultraviolet/visible) spectrophotometry, the drug-loading content and drug-encapsulation efficiency of MT in these NPs were determined to be 2.98% ± 0.16% and 80.12% ± 0.27%, respectively. Accordingly, we prepared PLGA/MT NPs coated with CXCR2-overexpressing macrophage membranes (CXCR2-MM) using a co-extrusion technique (Fig. 4A). Using transmission electron microscopy (TEM), we observed the spherical morphology of the PLGA and PLGA/MT NPs and the characteristic core–shell structure of the MM@PLGA/MT and CXCR2-MM@PLGA/MT NPs (Fig. 4F). Dynamic light scattering (DLS) analysis indicated that the average diameter of CXCR2-MM@PLGA/MT NPs was approximately 116.7 ± 2.0 nm, and the average ζ-potential and polydispersity index (PDI) were −30.8 ± 1.2 mV (Fig. 4G) and 0.225 ± 0.006, respectively. The completeness of the coating was confirmed using a biotinylated PLGA core that could be cross-linked in the presence of streptavidin (Fig. S5). After the ^Dil^CXCR2-MM@PLGA/MT NPs (containing Dil-labeled cell membranes) were centrifuged, the fluorescence intensity in the supernatant was markedly reduced, further indicating the high efficiency of membrane coating (Fig. S6). Notably, no marked change in particle diameter was detected when the CXCR2-MM@PLGA/MT NPs were immersed in DMEM containing 10% fetal bovine serum at room temperature, demonstrating the good colloidal stability of the suspension (Fig. S7). Furthermore, fluorescence microscopy images (Fig. 4H) demonstrated the overlap of PLGA NPs (green, labeled with coumarin6) with CXCR2-overexpressing membranes (red, labeled with DiR), indirectly confirming that the PLGA NPs were successfully coated with macrophage membranes. Western blot analysis further validated the presence of key membrane proteins—including CXCR2 and other critical receptors such as tumor necrosis factor receptor 2 [37] (TNFR2, a receptor for TNF-α), toll-like receptor 4 [38] (TLR4, a receptor for lipopolysaccharide), CD47 [39] (an immune checkpoint), and CD68 (a macrophage marker)—on both MM@PLGA and CXCR2-MM@PLGA NPs (Fig. 4I). The above-mentioned results indicated that the macrophage membranes encapsulating the NPs retained essential protein receptors, closely mimicking natural macrophage membranes. Additionally, CXCR2 expression was markedly higher in CXCR2-MM@PLGA NPs and RAW 264.7-CXCR2 cells than in MM@PLGA NPs and RAW 264.7 cells, respectively (Fig. 4I). These findings confirmed the successful synthesis of genetically engineered macrophage membrane-coated NPs.

We evaluated the drug release kinetics of CXCR2-MM@PLGA/MT, MM@PLGA/MT, and PLGA/MT NPs in a buffer simulating the extracellular environment (37 °C, PBS buffer, pH 7.4). After 48 h of incubation, the cumulative release rates of MT from PLGA/MT, MM@PLGA/MT, and CXCR2-MM@PLGA/MT were 66.4%, 61.8%, and 61.2%, respectively. Both MM@PLGA/MT and CXCR2-MM@PLGA/MT exhibited slightly slower MT release rates than PLGA/MT, but there was no marked difference between the MT release rates of CXCR2-MM@PLGA/MT and MM@PLGA/MT (Fig. 4J). Flow cytometry (FC) analysis demonstrated that the genetic modification for overexpressing CXCR2 did not impact the expression of CD11c and F4/80, which are key protein markers specific to macrophages (Fig. 4K). This indicated that the structural and functional integrity of the engineered macrophage cell membranes, which is critical for maintaining the biological efficacy of cell membrane-coated materials, was preserved during the preparation of CXCR2-MM@PLGA/MT NPs. Next, we investigated the effect of the engineered NPs on cell survival. Specifically, cytotoxicity was assessed in a hepatocyte cell line (AML12 cells) using the Cell Counting Kit-8 (CCK-8) assay. As shown in Fig. S8, comparisons with the control group revealed no marked changes in AML12 cell viability after 24 h of incubation with PLGA, MM@PLGA, and CXCR2-MM@PLGA NPs at concentrations of 10, 50, 100, and 200 μg/ml. These findings demonstrated the good cytocompatibility of PLGA NPs, macrophage membrane-coated NPs, and engineered CXCR2-expressing macrophage membrane-coated NPs.

Collectively, the results confirmed the successful synthesis and functional integrity of CXCR2-MM@PLGA/MT, which is necessary for achieving optimal biological effects. Furthermore, the results comprehensively supported the safety of these engineered NPs.

### Characterization of the immune evasion and chemokine adsorption capacity of CXCR2-MM@PLGA NPs in vitro

Most current drug delivery systems are cleared by the mononuclear phagocyte system before they can effectively reach their target site [40,41]. However, accumulating evidence suggests that macrophage membrane-coated NPs can escape phagocytosis by macrophages in vivo [42,43]. Therefore, we examined the uptake efficiency of PLGA, MM@PLGA, and CXCR2-MM@PLGA NPs in RAW 264.7 cells. To track cellular uptake, we labeled these NPs with coumarin6. As shown in Fig. S9A to D, strong green fluorescence was observed in the macrophages incubated with PLGA/coumarin6 for 4 h, demonstrating the marked uptake of PLGA NPs by RAW 264.7 cells. In contrast, minimal fluorescence was detected in macrophages incubated with MM@PLGA/coumarin6 and CXCR2-MM@PLGA/coumarin6. Furthermore, the overexpression of CXCR2 did not markedly alter the internalization of CXCR2-mm@PLGA/coumarin6 in macrophages, suggesting that CXCR2 overexpression does not enhance the immune evasion capacity of NPs.

We also evaluated the CXC chemokine adsorption capacity of CXCR2-MM@PLGA NPs in vitro. Our analysis showed that in murine IRI models, CXCL1 is predominantly expressed by hepatocytes, while CXCL2 is primarily produced by macrophages. To generate an in vitro IRI model, we subjected hepatocytes (AML12) and macrophages (RAW 264.7) to hypoxia, followed by reoxygenation. These cells were then co-incubated with PLGA, MM@PLGA, and CXCR2-MM@PLGA NPs, and the levels of CXCL1 and CXCL2 in the cell medium (CM) were measured (Fig. 4L). As shown in Fig. 4M, enzyme-linked immunosorbent assay (ELISA) revealed that CXCL1 and CXCL2 concentrations were markedly elevated in the hepatocyte and macrophage culture mediums after hypoxia–reoxygenation. Notably, the addition of PLGA and MM@PLGA NPs did not alter the CXCL1 and CXCL2 levels in the CM. However, the introduction of CXCR2-MM@PLGA NPs markedly reduced the levels of free CXCL1 and CXCL2 in the CM. Subsequently, we explored the in vitro effects of PLGA/MT NPs on signaling activity in AML-12 and RAW 264.7 cells. As shown in Fig. S10A to F, immunoblotting assays revealed that the incorporation of PLGA/MT NPs markedly inhibited the activation of the NF-κB pathway in AML-12 and RAW 264.7 cells while also suppressing the hypoxia–reoxygenation-induced up-regulation of CXC chemokine secretion. In contrast, PLGA NPs did not affect CXC chemokine levels or NF-κB pathway activity in these cells.

In summary, our results showed that coating PLGA NPs with macrophage membranes allows them to escape mononuclear phagocyte system clearance—a phenomenon that has been demonstrated and applied in a wide range of studies [42,43]. However, the overexpression of CXCR2 on cell membranes did not enhance this effect, suggesting that CXCR2 is unlikely to be involved in phagocytosis. Notably, macrophage membrane-coated NPs stand out for their ability to remove and neutralize pro-inflammatory cytokines [27]. Our study found that CXCR2-MM@PLGA NPs can markedly reduce the concentration of free CXCL1 and CXCL2 in the CM, suggesting that genetically engineered macrophage membrane-coated NPs may inhibit neutrophil chemotaxis by neutralizing CXCL1 and CXCL2. This validates our previous hypothesis that CXCR2 overexpression allows macrophage membrane-coated NPs to act as nanodecoys that target the CXCL–CXCR axis, trapping CXCL1 and CXCL2 within the inflammatory microenvironment to exert anti-inflammatory effects [32,44]. Finally, we explored the in vitro effects of PLGA/MT NPs and found that they were similar to the effects of MT in vitro, with both agents inhibiting CXC chemokine levels and suppressing NF-κB pathway activation in cells exposed to hypoxia–reoxygenation. These findings highlight the potential of genetically engineered macrophage membrane-coated drug delivery systems to resist immune clearance and generate anti-inflammatory effects.

### Targeting and treatment effects of CXCR2-MM@PLGA NPs in vivo

**Fig. 5.**
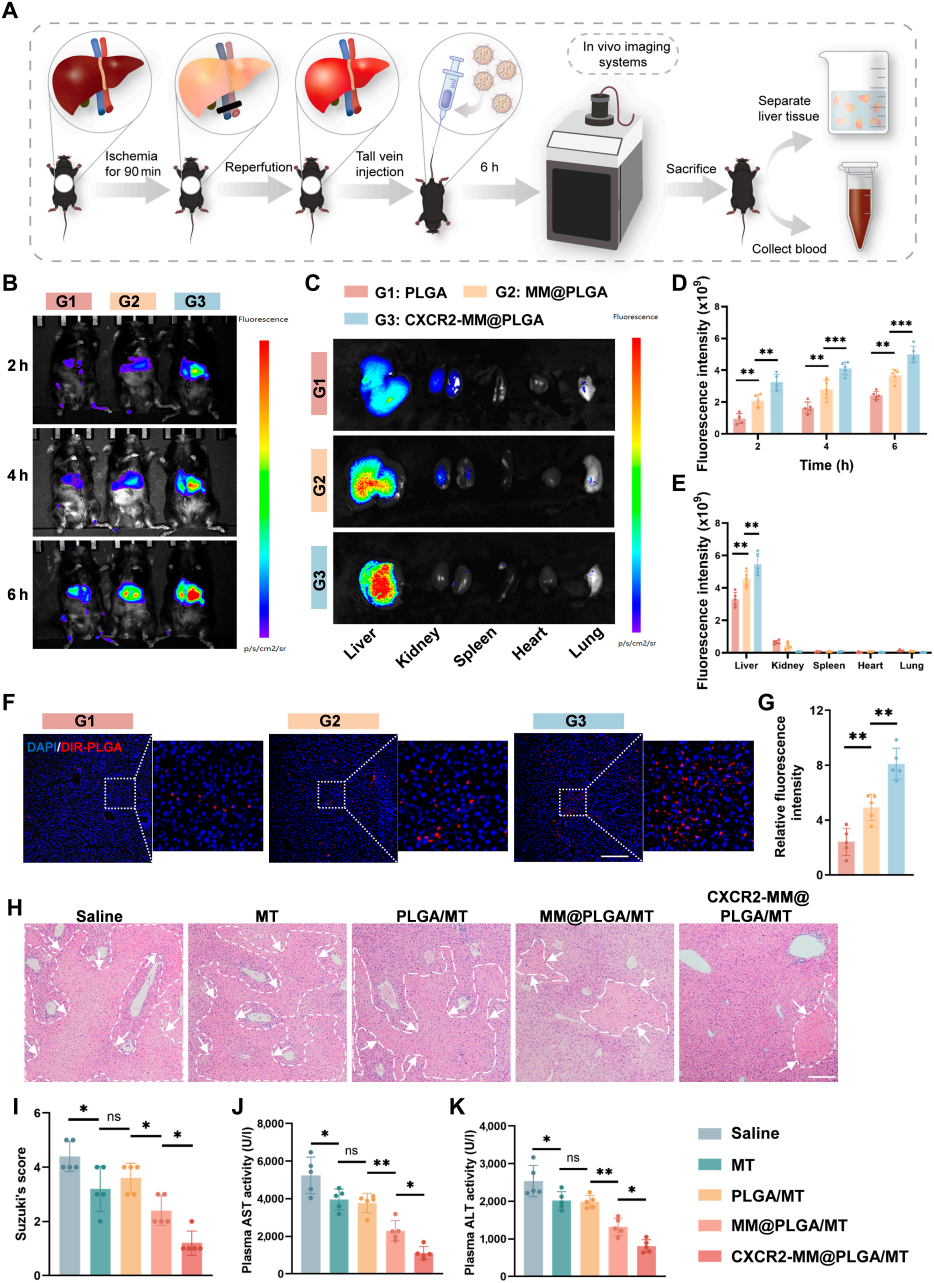
Engineered macrophage membrane-coated NPs enhance hepatic inflammatory targeting and attenuate HIRI. (A) Schematic representation of the in vivo treatment experiment. (B) Imaging of mice at various time points following hepatic ischemia–reperfusion after intravenous injection of PLGA/DiR, MM@PLGA/DiR, and CXCR2-MM@PLGA/DiR NPs (*n* = 5). (C) In vitro images of tissues (heart, liver, spleen, lungs, and kidneys) collected from hepatic ischemia–reperfusion mice after 6 h postinjection of PLGA/DIR, MM@PLGA/DIR, and CXCR2-MM@PLGA/DiR. (D) Quantitative analysis of nanoparticle fluorescence intensity in ischemia–reperfusion mouse livers at different time points (**P < 0.01, ***P < 0.001). (E) Quantification of fluorescence intensity in in vitro organ samples (**P < 0.01). (F) Representative fluorescence images showing the spatial distribution of CXCR2-MM@PLGA/DiR, MM@PLGA/DiR, and PLGA/DiR in ischemia–reperfusion livers of mice (*n* = 5). Scale bar = 200 nm. (G) Quantitative analysis of DiR fluorescence intensity in liver sections from different treatment groups (**P < 0.01). (H) Representative H&E-stained liver images from IRI mice treated with saline, MT, PLGA/MT, MM@PLGA/MT, or CXCR2-MM@PLGA/MT (*n* = 5). Scale bar = 200 nm. (I) Quantitative assessment of liver damage using Suzuki’s scoring system across treatment groups (“ns” denotes no significance, *P < 0.05). (J and K) ELISA results showing serum ALT and AST levels in different groups (*n* = 5) (“ns” denotes no significance, *P < 0.05, **P < 0.01).

We proceeded to investigate the effect of CXCR2-MM@PLGA NPs on HIRI in vivo, as outlined in Fig. 5A. A partial hepatic thermal ischemia–reperfusion model was established as described previously [45]. To assess NP targeting and accumulation in vivo, we labeled PLGA NPs with DiR. We used an in vivo imaging system to track the distribution of PLGA/DiR, MM@PLGA/DiR, and CXCR2-MM@PLGA/DiR NPs at various time points after tail vein injection. As shown in Fig. 5B and D, in the PLGA/DiR group, fluorescence signals in the liver were minimal. In contrast, the MM@PLGA/DiR group displayed enhanced hepatic DiR signals. Furthermore, CXCR2-MM@PLGA/DiR NPs demonstrated better targeting of inflammatory sites than MM@PLGA/DiR NPs. Subsequently, we examined the organ distribution of the different NPs in the mouse models. At 6 h postinjection, the livers and major organs of the mice were harvested to measure fluorescence intensity. As shown in Fig. 5C and E, the CXCR2-MM@PLGA/DiR fluorescence signal in the liver was markedly higher than the MM@PLGA/DiR and PLGA/DiR fluorescence signal, indicating that CXCR2 overexpression on macrophage membranes enhances the ability of NPs to target sites of inflammatory damage. Notably, MM@PLGA/DiR and CXCR2-MM@PLGA/DiR exhibited low fluorescence signals in other organs. In contrast, PLGA/DiR NPs showed the fluorescence signals in the kidneys. These findings indicated that non-cell membrane-coated NPs were rapidly cleared by the immune system in mice. Moreover, MM@PLGA/DiR and CXCR2-MM@PLGA/DiR showed similar fluorescence intensities in other organs such as the kidneys, suggesting that CXCR2 overexpression does not enhance the ability of cell membrane-coated NPs to evade immune clearance, consistent with our in vitro findings. To further confirm this, we performed fluorescence staining and assessed the accumulation of different NPs in the central portion of the liver. As shown in Fig. 5F and G, the PLGA/DiR group displayed weak fluorescence in the region of liver reperfusion injury, likely due to poor targeting and rapid immune clearance. In contrast, CXCR2-MM@PLGA/DiR showed markedly greater accumulation at the site of injury than MM@PLGA/DiR.

After determining the targeting capacity of CXCR2-MM@PLGA/MT, we evaluated the therapeutic efficacy of these NPs in vivo. After removing the microvascular clamp and restoring blood flow for 5 min, saline, MT, PLGA/MT, MM@PLGA/MT, and CXCR2-MM@PLGA/MT were administered via tail vein injection. The presence of liver injury after reperfusion was assessed using hematoxylin and eosin (H&E) staining, as shown in Fig. 5H and I. Compared to the saline group, the MT and PLGA/MT groups showed a modest but similar decrease in hepatic tissue injury. In contrast, the MM@PLGA/MT group exhibited a marked reduction in liver damage and hepatocellular necrosis, while the extent of liver injury was the smallest in the CXCR2-MM@PLGA/MT group. This indicated that CXCR2-MM@PLGA/MT provided the strongest protective effect against HIRI. These results were further validated by the findings from serum assays. Compared to the saline group, the MT and PLGA/MT groups showed a slight reduction in serum alanine transaminase (ALT) and aspartate transaminase (AST) levels. However, a marked decrease in these levels was observed in the MM@PLGA/MT and CXCR2-MM@PLGA/MT groups (Fig. 5J and K). The CXCR2-MM@PLGA/MT group exhibited the most pronounced reduction in serum ALT and AST levels, thus providing the best protective effect.

In summary, our experimental results indicated that CXCR2-MM@PLGA NPs can effectively target sites of HIRI inflammation. This could be attributed to the ability of macrophage membranes to confer PLGA NPs with immune evasion capabilities and the effect of cell membrane CXCR2 overexpression, which can enhance the accumulation of NPs at HIRI sites by targeting the CXCL–CXCR axis [30,46]. These improvements increased the targeting efficiency of PLGA NPs, potentially inhibiting the inflammatory cascade in the early stages of HIRI. In addition, we found that CXCR2-MM@PLGA/MT was effective at attenuating liver injury in mouse models of HIRI. This was because these NPs showed an enhanced ability to target the sites of HIRI, enabling more efficient drug delivery and thus attenuating injury. These results confirm the targeting and therapeutic effectiveness of CXCR2-MM@PLGA/MT in mitigating liver injury in mouse models of HIRI, demonstrating the in vivo efficacy of these NPs.

### In vivo anti-inflammatory activity of CXCR2-MM@PLGA NPs

**Fig. 6.**
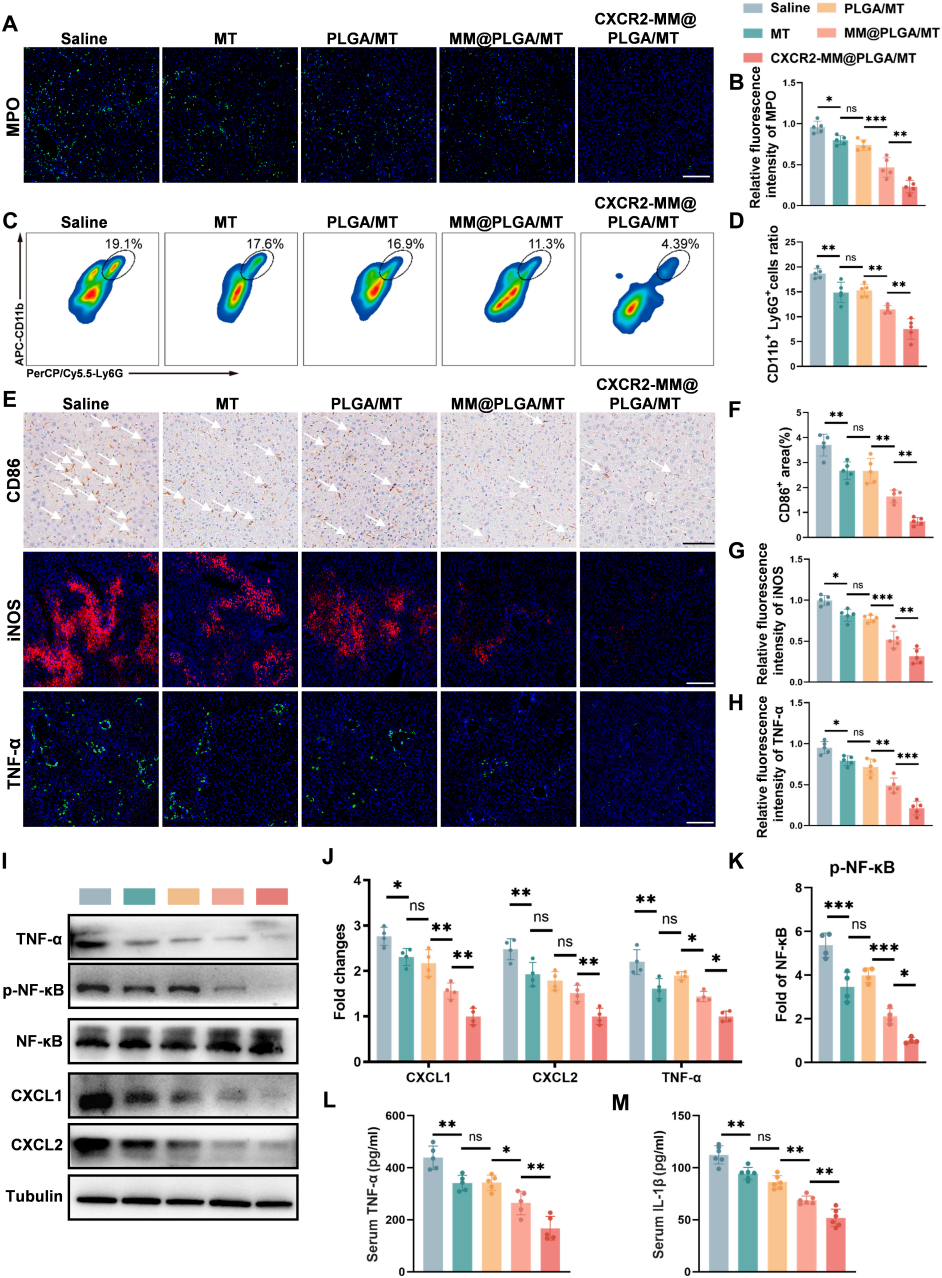
CXCR2-MM@PLGA/MT attenuates HIRI in vivo by inhibiting macrophage pro-inflammatory polarization and neutrophil infiltration. (A) Immunofluorescence staining of MPO in different IRI mice groups (*n* = 5). Scale bar = 200 nm. (B) Quantitative analysis of MPO^+^ cells from immunofluorescence images (“ns” denotes no significance, *P < 0.05, **P < 0.01, ***P < 0.001). (C) Flow cytometry analysis of neutrophil infiltration in different IRI mice groups, with neutrophils labeled using APC-CD11b and PerCP/Cy5.5-Ly6G. (D) Quantitative analysis of neutrophil infiltration based on flow cytometry results (“ns” denotes no significance, **P < 0.01). (E) Immunofluorescence staining for iNOS and TNF-α (scale bar = 200 nm), and immunohistochemical staining for CD86 (scale bar = 100 nm) in different IRI mice groups (*n* = 5). (F to H) Quantitative analysis of the fluorescence intensity of iNOS^+^ and TNF-α^+^ cells, as well as the positive area of CD86 (“ns” denotes no significance, *P < 0.05, **P < 0.01, ***P < 0.001). (I) Representative Western blot images showing hepatic iNOS, TNF-α, p-NF-κB, NF-κB, CXCL1, and CXCL2 protein expression in different IRI mice groups. (J and K) Quantitative analysis of the Western blot results, with tubulin as a loading control (“ns” denotes no significance, *P < 0.05, **P < 0.01, ***P < 0.001). (L and M) Serum concentrations of inflammatory cytokines in different IRI mice groups, measured by ELISA (*n* = 5) (“ns” denotes no significance, *P < 0.05, **P < 0.01).

CXCR2-MM functions as a nanodecoy for CXC chemokines, and its ability to sequester CXCL1 and CXCL2 was confirmed in our in vitro experiments. To further investigate whether CXCR2-MM can elicit similar anti-inflammatory effects in vivo, we examined neutrophil infiltration and inflammatory factor levels following HIRI through immunostaining assays for myeloperoxidase (MPO), a neutrophil marker [47], and tumor necrosis factor-α (TNF-α), a key inflammatory cytokine [48]. As shown in Fig. S11A to C, TNF-α fluorescence signals were the strongest in the control group and the PLGA group. In contrast, the MM@PLGA and CXCR2-MM@PLGA groups showed a marked reduction in the region of TNF-α fluorescence. In addition, MPO^+^ cells were widely distributed at the site of injury in the control, PLGA, and MM@PLGA groups. In contrast, the number of infiltrating neutrophils within the central region of HIRI was markedly reduced in the CXCR2-MM@PLGA group. In line with these findings, FC revealed that the ratio of CD11b^+^ to Ly6G^+^ cells was the lowest in the CXCR2-MM@PLGA group (Fig. S9D and E). Subsequently, we investigated whether CXCR2-MM@PLGA/MT elicits anti-inflammatory effects in vivo. As shown in Fig. 6A and B, MPO^+^ cells were widely distributed at the site of injury in the saline-treated group. Meanwhile, the MT and PLGA/MT groups showed minimal reductions in the number and area of MPO^+^ cells. Interestingly, although a marked reduction in both metrics was seen in the MM@PLGA/MT group, the CXCR2-MM@PLGA/MT group showed the highest reduction in the number of infiltrating neutrophils within the central area of HIRI. FC analysis of reperfusion-treated liver tissues revealed that the percentage of CD11b^+^/Ly6G^+^ cells was only modestly reduced in the MT and PLGA/MT-treated groups when compared to the saline-treated group. In contrast, the MM@PLGA/MT-treated group exhibited a more pronounced decrease in CD11b^+^/Ly6G^+^ cell infiltration, and the CXCR2-MM@PLGA/MT-treated group had the lowest percentage of CD11b^+^/Ly6G^+^ cells (Fig. 6C and D). These results were consistent with the immunostaining outcomes, further highlighting that CXCR2 overexpression on macrophage membranes can effectively inhibit peripheral neutrophil infiltration following HIRI by targeting the CXCL–CXCR chemokine axis.

To investigate whether genetically engineered macrophage membrane-coated NPs can ameliorate the pro-inflammatory phenotype of macrophages in vivo, the expression of the M1 markers iNOS and CD86 was tested. Immunofluorescence analysis revealed the widespread distribution of iNOS^+^ cells in the saline group, indicating that the macrophages predominantly adopted the M1 pro-inflammatory phenotype following ischemia–reperfusion in this group (Fig. 6E and G). The MT and PLGA/MT groups exhibited a slight reduction in the number of iNOS^+^ cells. However, the MM@PLGA/MT group showed a marked reduction in both the number and area of iNOS^+^ cells, with the CXCR2-MM@PLGA/MT group providing an even more pronounced reduction. This conclusion was further supported by CD86 immunohistochemistry assays. While the number of CD86^+^ cells was largely unchanged following MT and PLGA/MT treatment, MM@PLGA/MT substantially reduced CD86^+^ cell infiltration. Notably, the CXCR2-MM@PLGA/MT group demonstrated the lowest infiltration of CD86^+^ cells (Fig. 6E and F). Next, we explored whether genetically engineered macrophage membrane-coated NPs could modulate inflammatory factor levels and chemokine expression in vivo following HIRI. Our results demonstrated that the fluorescence signal for TNF-α was the most prominent in the saline group, followed by the MT and PLGA/MT groups. In contrast, MM@PLGA/MT treatment led to a marked decrease in the area of TNF-α fluorescence, and CXCR2-MM@PLGA/MT yielded the most pronounced effect, leading to the weakest TNF-α fluorescence intensity (Fig. 6E and H). These findings were further corroborated by Western blot analysis, which showed a slight decrease in TNF-α expression following MT and PLGA/MT treatment and a more marked reduction following MM@PLGA/MT treatment. However, the greatest reduction in TNF-α protein levels was observed after CXCR2-MM@PLGA/MT treatment, achieving the greatest reduction of inflammatory markers (Fig. 6I and J). Interestingly, the expression of the chemokines CXCL1 and CXCL2 followed a similar trend. The ratio of phosphorylated NF-κB (p-NF-κB) to total NF-κB was slightly reduced in the MT and PLGA/MT group, but more markedly reduced in the MM@PLGA/MT group. Notably, the most effective suppression of the p-NF-κB to total NF-κB ratio was detected in the CXCR2-MM@PLGA/MT group (Fig. 6I to K). These results were consistent with our in vitro findings, suggesting that CXCR2-MM@PLGA/MT NPs can inhibit neutrophil recruitment and tissue inflammation in vivo*.* To further validate these observations, we employed ELISA and quantitatively assessed the serum levels of the pro-inflammatory cytokines TNF-α and IL-1β in mice with HIRI. The MT and PLGA/MT groups showed a moderate reduction of TNF-α and IL-1β serum concentrations, while the MM@PLGA/MT group showed a greater decrease. Notably, the CXCR2-MM@PLGA/MT group achieved the most marked reduction in pro-inflammatory cytokine levels among all groups (Fig. 6L and M).

In summary, our in vivo experiments showed that CXCR2 overexpression on macrophage membranes can reduce post-HIRI inflammation while inhibiting neutrophil infiltration. This was attributed to the retention of inflammation-related receptors on the macrophage cell membrane and the artificial overexpression of CXCR2, which inhibited inflammatory responses and neutrophil recruitment by adsorbing cytokines and chemokines at the site of injury [44,49]. Moreover, CXCR2-MM@PLGA/MT NPs were more effective at attenuating post-HIRI injury and modulating macrophage inflammation to prevent M1-type polarization. The overexpression of CXCR2 also enhanced the ability of the NPs to specifically target the site of inflammation [50], enabling more efficient drug delivery and cytokine/chemokine adsorption, thereby reducing the inflammatory response and inhibiting neutrophil infiltration. These findings highlight the potential of CXCR2-overexpressing cell membrane-coated NPs in mitigating inflammation. Overall, our study demonstrates the potential of CXCR2-MM@PLGA/MT as a promising therapeutic tool against HIRI, as these NPs effectively inhibit the chemotaxis of peripheral inflammatory cells and suppress local inflammation in vivo.

### Biosafety of CXCR2-MM@PLGA NPs

**Fig. 7.**
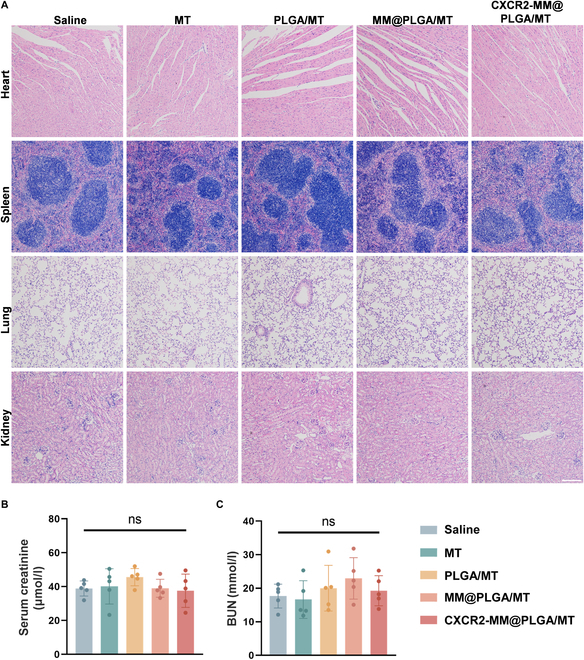
CXCR2-MM@PLGA/MT exhibits excellent in vivo biosafety. (A) H&E staining of major organs, including the heart, spleen, lungs, and kidneys in different IRI mice groups (“ns” denotes no significance). Scale bar =200 nm. (B and C) Biochemical analysis of renal function markers, including blood creatinine and blood urea nitrogen levels (“ns” denotes no significance, *n* = 5).

Finally, the biosafety of CXCR2-MM@PLGA NPs was thoroughly evaluated in this study. After the treatment period was complete, major organs—including the heart, spleen, lungs, and kidneys—were collected from the different groups of mice and analyzed histologically to assess potential toxicological effects. As shown in Fig. 7A, no marked damage or abnormalities were observed using H&E staining. Additionally, serum markers of renal function, such as creatinine and blood urea nitrogen, revealed no indications of systemic toxicity in any treatment group (Fig. 7B). These findings suggested that PLGA NPs coated with engineered macrophage membranes exhibit a favorable biosafety profile. Collectively, the findings highlighted the biocompatibility of these functionalized NPs, underscoring their potential for future biomedical applications.

## Conclusion

This study underscores the critical role of CXC chemokines in HIRI and reveals their cellular origins using bioinformatics analysis. Our results demonstrated that MT reduces the phosphorylation of NF-κB in both macrophages and hepatocytes, thereby inhibiting the synthesis and secretion of CXC chemokines. Building on these results, we developed CXCR2-MM@PLGA/MT as an innovative therapeutic platform for HIRI. These NPs not only retained the functional properties of previously developed macrophage membrane-coated NPs, such as the ability to scavenge pro-inflammatory cytokines and escape immune clearance, but also specifically enhanced the ability of the NPs to scavenge the key inflammatory mediators of HIRI owing to the overexpression of CXCR2. Moreover, these NPs showed improved targeting ability and enhanced the accumulation of MT at the site of inflammation. Accordingly, they inhibited the activation of pro-inflammatory pathways and NF-κB signaling in hepatocytes and macrophages through the release of MT, effectively down-regulating inflammatory factors. Overall, these genetically engineered macrophage membrane-coated NPs reduced neutrophil infiltration and mitigated the secondary inflammatory cascade associated with HIRI. Therefore, the engineered cellular NP-based therapy developed in this study could serve as a safe, effective, and cost-efficient strategy for treating HIRI.

## Ethical Approval

All experiments complied with ethical regulations and were approved by the Animal Ethical and Experimental Committee of the Chongqing Medical University.

## Data Availability

Data will be made available on a reasonable request.

## Supplementary Materials

Supplementary 1 Supplementary Section Table S1 Figs. S1 to S11
