# Supplementary material for: Engineered Macrophage Membrane-Coated Nanoparticles for Hepatic Ischemia–Reperfusion Injury Therapeutics
Source: Biomater Res. 2025 May 23;29:0212. doi: 10.34133/bmr.0212 (PMC12099054; doi:10.34133/bmr.0212)
Supplement: Supplementary 1 — Supplementary Section Table S1 Figs. S1 to S11 [file bmr.0212.f0212.doc]

Experimental Section

*Nanoparticle Physicochemical Characterization:* The size distribution and zeta potential of the nanoparticles (NPs) were determined using NanoBrook (Brookhaven Instruments, USA). The morphology of the NPs was observed using transmission electron microscopy (Hitachi, Japan). In DMEM containing 10% fetal bovine serum after incubation at room temperature for different times to evaluate the serum stability of the NPs. To confirm the successful coating of macrophage membranes on the surface of PLGA NPs, we labelled the membranes with DiR. Subsequently, DiR-labelled membranes were coated on PLGA/coumarin6 NPs using a direct extrusion method, as previously described^[44]^ to obtain dual fluorescent labelled mimetic NPs (CXCR2-MM/DIR@PLGA/coumarin6). The levels of CXCR2 and other cell membrane marker proteins in PLGA, MM@PLGA, CXCR2-MM@PLGA, RAW264.7 cells and RAW264.7-CXCR2 cells were detected by immunoblotting analyses. Dil-stained CXCR2-RAW 264.7 cell membranes (^Dil^CXCR2-MM) were prepared by mixing cell membranes with Dil at a membrane protein/dye weight ratio of 1000:1. Nanoparticles were prepared using PLGA cores containing 5% biotinylated PLGA (BP-29254, BroadPharm) at different membrane coating ratios. The size was measured using DLS before and after 1 h of incubation with 10 µg/ml streptavidin (Beyotime).

## *Drug loading and in vitro drug release study:* PLGA/MT, MM@PLGA/MT, and CXCR2-MM@PLGA/MT NPs were first frozen at -80 ℃ and then freeze-dried using a Labconco Free Zone lyophilizer. The lyophilized NP powder was dissolved in DMSO, and the absorbance was measured using a UV/Vis spectrophotometer (DU730, Beckman Coulter) at 222 nm. According to the established standard curve of MT in DMSO, the drug-loading content and drug-encapsulation efficiency were calculated as follows:

$$loading content\left( \% \right)=\frac{M_{MT}}{M_{PLGA}+M_{MT}}\times100\%$$

$$encapsulation efficiency\left( \% \right)=\frac{M_{MT}}{M_{added}}\times100\%$$

where *M_MT_* is the mass of MT loaded in the NPs, *M_PLGA_* is the mass of polymer in the formulation and *M_added_* is the mass of MT added.

The drug (MT) release from PLGA/MT, MM@PLGA/MT, and CXCR2-MM@PLGA/MT NPs was studied separately using a dialysis method. Briefly, PLGA/MT, MM@PLGA/MT, and CXCR2-MM@PLGA/MT NPs solutions were added to disposable dialysis bags (3.5 kDa MWCO, Millipore Sigma). The dialysis bags were then immersed in 10 mL of phosphate-buffered saline (PBS) solution (release medium, pH 7.4) at 37 ℃. Three independent replicates were used for each sample. One milliliter of release medium was collected for analysis at different time intervals and replaced with an equivalent volume of fresh PBS at 37 ℃. The cumulative amount of released MT was quantified using a UV/Vis spectrophotometer (DU730, Beckman Coulter) at 222 nm.

*Public data collection and differential gene analysis:* Transcriptome data and single-cell data were obtained from the Gene Expression Omnibus (GEO) for gene ontology (GO) enrichment analysis, and the sequence numbers of the data used were GSE151648 and GSE189539. The Wilcoxon test was performed using the wilcox.test function to screen for genes with a 4-fold or greater difference in gene expression between the experimental and control groups (log2 Fold Change = 2); P<0.05, indicating significant difference, and the resulting genes were differentially expressed genes. The sequence numbers of the single-cell sequencing data were GSE189539 (human) and CRA004061, GSE137869 (murine). The data used for the ROC curves were GSE151648. The online website (https://cloud.oebiotech.com/) was used to visualize the results in the data section, and the remaining data manipulation was performed using R (V4.2.1).

*Quantitative Reverse Transcription PCR (RT-qPCR) Measurement:* Total RNA was extracted from treated cells and tissues with Trizol (Absin) and then reverse-transcribed into cDNA using the PrimeScript RT kit (Takara). *RT-qPCR* was performed using the SYBR Green qPCR Master Mix (MCE). Cycling conditions were 95℃ for 1 min, followed by 40 cycles of 95℃ for 15 s, followed by 60℃ for 45 s. The relative expression of the target genes was calculated using β-actin as an internal reference gene. The primer sequences used were as follows:

CXCL1 (forward: 5'-ACTCAAGAATGGTCGCGAGG-3′. reverse: 5′-ACTTGGGGACACCTTTTAGCA-3′)

CXCL2 (forward: 5'-CACTCTCAAGGGCGGTCAAA-3′. reverse: 5′-CAGTTAGCCTTGCCTTTGTTCA-3′)

CXCR2 (forward: 5'-GGGTCGTACTGCGTATCCTG-3′. reverse: 5′-AGACAAGGACGACAGCGAAG-3′)

TNF-α (forward: 5'-TAGCCCACGTCGTAGCAAAC-3′. reverse: 5′-GCAGCCTTGTCCCTTGAAGA-3′)

β-actin (forward: 5'-GGCTGTATTCCCCTCCATCG-3′. reverse: 5′-CCAGTTGGTAACAATGCCATGT-3′)

*Cellular Immunofluorescence：*According to previous study^[30]^. Cellular immunofluorescence was used to detect the expression of CXCR2 on the surface of RAW 264.7 cells. Briefly, RAW 264.7, cells were inoculated in 24-well plates at a density of 1 × 10^5^ cells/well. After the indicated treatments, cells were washed with PBS, fixed with 4% paraformaldehyde for 15 min, and permeabilized with 0.2% TritonX-100 for 30 min. After incubation in 5% BSA for 1 h, cells were stained with CXCR2 monoclonal antibody (1:100) overnight in the dark. After washing with PBS, the cells were incubated with Dylight549-coupled secondary antibody (1:200) for 2 h at room temperature in the dark. The cell nuclei were stained with DAPI for 10 min. After washing with PBS, the immunofluorescence images were captured using an immunofluorescence microscope (Nikon Ti2).

*Cytotoxicity test:* The CCK-8 assay was used to evaluate cell viability *in vitro*. Specifically, AML12 cells were inoculated into 96-well culture plates at a density of 1 × 10^4^ cells per well. Adherent cells were co-incubated with different concentrations of the material for 48 h, and cell viability was assessed using CCK-8 (Beyotime). Absorbance values were measured at 450 nm using an enzyme marker (VARIOSKAN LUX, Thermo Fisher Scientific).

*Endocytosis assay：*To assess the effect of macrophage membrane camouflage on macrophage phagocytosis, we performed experiments using RAW264.7 macrophages. Macrophages were assayed for the internalization of PLGA/coumarin6, MM@PLGA/coumarin6 and CXCR2-MM@PLGA/coumarin6. Briefly, RAW264.7 macrophages were inoculated in 24-well plates at a density of 5 × 104 cells/well and cultured for 24 h. PLGA/coumarin6, MM@PLGA/coumarin6 and CXCR2-MM@PLGA/coumarin6 were added(100ug/ml PLGA/coumarin6 per well). After incubation for 0.5, 1, 2, and 4 h, the cell nuclei were stained with DAPI for fluorescence imaging.

*Flow cytometry:* Flow cytometry was used to detect the expression of CXCR2, F4/80, CD11b, Ly6G and CD11c in samples such as RAW 264.7 cells, RAW 264.7-CXCR2 cells, CXCR2-MM@PLGA and liver tissue. Briefly, samples were incubated with PE anti-mouse CXCR2 antibody, APC anti-mouse F4/80 antibody, APC anti-mouse CD11b antibody, PerCP/Cy5.5 anti-mouse Ly6G, and PE anti-mouse CD11c antibodies (Biolegend, USA) for 30 min in the dark at 4℃. Subsequently, the specimens were washed with PBS, collected by centrifugation (3000 rpm, 4℃, 5 min), and analyzed using flow cytometry (FACSVerse 8, BD, USA).

*Cell hypoxia-reoxygenation (H/R) model：*Timing of H/R and methods based on previous studies^[25]^*.* RAW264.7, and AML12 cells were cultured under normal oxygen concentrations. To establish the H/R model, cells were placed in a three-gas incubator, and the parameters were adjusted to oxygen 1%, carbon dioxide 5%, and nitrogen 94% to construct hypoxic cell culture conditions. The parameters were adjusted to 20% oxygen, 5% carbon dioxide, and 75% nitrogen after 3 h of anoxic incubation to restore the normoxic cell culture conditions. cells were collected after 6 h for subsequent experiments.

*Animal model of HIRI：*According to previous study^[45]^. Animal models of ischemia-reperfusion injury were established using C57BL/6 mice (male, 8-10 weeks). Prior to surgery, the mice were fasted for 12 h, but had free access to water. During surgery, the mice were fully anesthetized and a constant temperature was maintained using a heating blanket. These mice were immobilized on an operating table and underwent caesarean section to expose the liver. The hepatic artery and portal vein were blocked using sterilized clamp, resulting in deprivation of approximately 70 % of blood flow to the liver. After 90 min of partial ischemia, the clamp was removed to initiate hepatic reperfusion, and the abdominal incision was closed with sutures. In the treatment groups, Free MT, PLGA/MT, MM@PLGA/MT or CXCR2-MM@PLGA/MT in saline was administered to the mice (1 mg/kg MT each group). PLGA, MM@PLGA, or CXCR2-MM@PLGA in saline was administered to the mice (5 mg/kg PLGA each group). Mice treated with saline alone were used as the control group. All animal experiments were approved by the Institutional Animal Ethics Committee of Chongqing Medical University.

*Histological and immunofluorescence analysis：*Liver tissues were paraffin-embedded, sectioned to 8-10 μm and stained with hematoxylin and eosin (H&E). The expression of MPO, iNOS, and TNF-α was detected by immunofluorescence. Briefly, after blocking, tissue sections were incubated with primary antibodies against MPO, iNOS, and TNF-α overnight at 4℃. After washing with PBS, fluorescent secondary antibodies were added and the cells were incubated for 50 min at room temperature, followed by restaining with DAPI. Images of the sections were captured using a fluorescence microscope (Nikon Ti2) and quantified using ImageJ software. Immunohistochemistry (IHC) was performed to detect CD86 expression. Briefly, after blocking, tissue sections were incubated with primary antibody against CD86 overnight at 4℃. After washing with PBS, the fluorescent secondary antibody was added and incubated for 50 min at room temperature, followed by chromatography with DAB (Solarbio), and finally stained with hematoxylin (Beyotime). Images of the sections were captured using a light microscope (Leica) and quantified using the ImageJ software.

*Enzyme-linked immunosorbent assay (ELISA)：*To assess the expression of CXCL1 and CXCL2 in cell supernatants and the expression levels of the pro-inflammatory cytokines TNF-α and IL-1β in the serum of ischemia-reperfusion mice. RAW 264.7, AML12 cells were seeded in 6-well plates at 2 × 10^5^ cells per well and cultured for 24 h. PLGA, MM@PLGA, and CXCR2-MM@PLGA were added (100ug/ml PLGA per well) and the cells were then treated with hypoxic-reoxygenation. After co-incubation, the NPs were removed by centrifugation at 15000 × g for 15 min and the supernatant was collected. The supernatant cultures of AML12 and RAW264.7 cells treated with NPs, and the sera of ischemia-reperfusion mice treated with different groups were collected, and the concentrations of cytokines were measured using ELISA kits (Jiubang Biotechnology, China). The OD values were determined using an absorbance enzyme marker (VARIOSKAN LUX, Thermo Fisher Scientific), and the concentrations were calculated from the standard curve.

*In vivo imaging：*For in vivo imaging, C57BL/6 mice were randomly divided into 3 groups:(1) PLGA/DIR, (2) MM@PLGA/DIR, and (3) CXCR2-MM@PLGA/DIR). An equal quantity of NPs was injected intravenously through the tail vein after ischemia-reperfusion (2 mg/kg PLGA/DIR each group). The in vivo distribution was imaged at different time intervals using an AniView Pro system (USA) with an excitation wavelength of 748 nm and emission wavelength of 780 nm. In addition, mice were euthanized and their major organs were removed. The fluorescence intensities of the heart, liver, spleen, kidney, and lung were also measured to assess the in vitro distribution of NPs.

*In vivo toxicity evaluation：*Toxicity assessment and *in vivo* histopathological studies were performed after treatment. Organic specimens (heart, spleen, lungs, and kidneys) were collected from saline, PLGA/MT, MM@PLGA/MT and CXCR2- MM@PLGA/MT group mice for sectioning. All specimens were subsequently stained with hematoxylin and eosin using an H&E staining kit (Beyotime) and examined histopathologically. In addition, blood samples were collected from different groups of experimental animals for evaluation of renal BUN and serum creatinine (Solarbio) function using biochemical analysis.

*Statistical Analysis：*The Statistical analysis of the experimental results was conducted using GraphPad Prism 6. The data are expressed as the mean ± standard deviation (s.d.) from at least three independent experiments (n ≥ 3). Student’s t-test was used for comparisons between two groups, and a P-value of less than 0.05 was regarded as statistically significant. When comparing multiple experimental groups, a two-way ANOVA statistical test was performed.

**Supplementary Table S1.** Details on the antibodies used in the present study

| **Antibodies** | **Source** | **Dilution** | **Catalogue number** | **Manufacturer** |
| --- | --- | --- | --- | --- |
| Anti-CXCR2 antibody | Rabbit | WB: 1:1500  IF:1:200 | A3301 | abclonal |
| Anti-CXCL1 antibody | Rabbit | WB:1:500 | A25014 | abclonal |
| Anti-CXCL2 antibody | Rabbit | WB:1:500 | A12639 | abclonal |
| Anti-p-NF-κB antibody | Rabbit | WB: 1:500 | AP0124 | abclonal |
| Anti-NF-κB antibody | Rabbit | WB: 1:1500 | ET1604 | Huabio |
| Anti-β-actin antibody | Mouse | WB: 1:1500 | 66009-1-Ig | Proteintech |
| Anti-TLR4 antibody | Rabbit | WB: 1:500 | 19811-1-AP | [Proteintech](https://www.baidu.com/link?url=mqXL0JCE06yhgCXA8oRyqazqrlpgr-_mxtm-hnTS0q7&wd=&eqid=f40bddd0000145c60000000662383f86) |
| Anti-TNFR2 antibody | Rabbit | WB: 1:1500  IHC:1:200 | A24513 | abclonal |
| Anti-CD68 antibody | Rabbit | WB: 1:1500 | 28058-1-AP | Proteintech |
| Anti-iNOS antibody | Rabbit | WB: 1:1500  ICC:1:200 | Ab178945 | Abcam |
| Anti-TNF-α antibody | Rabbit | WB: 1:1500 | 17590-1-AP | Proteintech |
| Anti-TNF-α antibody | Rabbit | ICC: 1:200 | GB115701 | [Servicebio](https://www.baidu.com/link?url=mqXL0JCE06yhgCXA8oRyqazqrlpgr-_mxtm-hnTS0q7&wd=&eqid=f40bddd0000145c60000000662383f86) |
| Anti-Myeloperoxidase antibody | Rabbit | ICC: 1:200 | Ab208670 | Abcam |
| PE Anti-CXCR2 antibody | Rat | FC: 1:100 | 149609 | Biolegend |
| APC Anti-CD11c antibody | Rat | FC: 1:100 | 117309 | Biolegend |
| APC Anti-F4/80 antibody | Rat | FC: 1:100 | 2691600 | invitrogen |
| Anti-CD86 antibody | Rabbit | IHC: 1:100 | 91882 | [Cell Signaling Technology](http://www.baidu.com/baidu.php?url=af0000KpxzUee8Wytj49nclCwHld_LmOz-6yOK-0nE9O-txhpPARiVdNrdfpc0lrOm_attPcbWU3Hjhw_Dqz22xNz8042HIVSpwzoieQIq1GR4P-6SefRowLhuYcDxeaMtetQCy8EE3KzeBxiWGQqw73jNIopHWQsz6B22imR-q9GgWggsuFUAUQveKt7NFDINk42UXgI0SMyrUxDTIrBnG0S5o7.7b_NR2Ar5Od66xAS6MzEukmDfwECF63nEjspPZWuKuu_zUeVOZ5jod_4S1c1o1ecmyTSEu3e81_LI-xu88eltHDLeqOYxhOx9emJ3UCJq5ZtvyOVhOxdkkulZUSUQGeINxzlsdnIMySe-SWDkPSOxqZ4EL3Xv28Al2EHkOP-EeuELqTTyX9vOhzlXLNg3qDgeqbXJE6lFEL3xCBo9SWtLexS8etLeOSEojRkvIUe8yOCWCnXUvxOKWOBNOkQnMoDOWOVgxSjgtE-u3OxO_xtarZxISLslUceQQQ51uxqIv8zzzzzzzzzzz1GBSg4E-SUqOlSnNOSEqX8Ex-dLOQ_BzetE4oOJ1suxOKtCEvOE-BolXZggugIfCxxq8loWsyfvyyuxLOLOz3SjkdvyyxgOsX1CO21q7---brVMO_yTZLOUsprL-9ObxhORsxwvUOqMv4qhj9ue5qJ9_OZuqO3M4Ekz4OvtAWV4mx4Z4EdnZYIhOOuq8xu63yX1OpCU5kvxxSFSUtx1uDSx4txOmOZ-zOtg4Oq-MdO3E519OCkUr19lZHF3xCCxfMqEVO3EY5CHe5Y5qMZOkT_I----IdOZi_HAOuuguu_LtVvGmuCy2S5Hk_R.U1Yk0ZDqfdPR0ZKGm1Yk0ZfqmvN_UaRznZPGuv49UA-8uzRznQhozel4vTL30A-V5HDYPWD0u1dsT1c0Iybqmh7GuZN_UfKspyfqn1n0mv-b5Hf1P6KVIjY3PWc4g1DsnHIxnH0krNt1PW0k0AVG5H00TMfqrjRd0AFG5HDdr7tznjwxPH010AdW5HKxnH0kPdtknjD4g100TgKGujYs0Z7Wpyfqn0KzuLw9u1Ys0A7B5HKxn0K-ThTqn0KsTjYs0A4vTjYsQW0snj0snj0s0AdYTjYs0AwbUL0qn0KzpWYs0Aw-IWdsmsKhIjYs0ZKC5H00ULnqn0KBI1Yz0A4Y5H00TLCq0A71gv-bm1dsTzd-mvC0pywW5R420ZKCIZbq0Zw9ThI-IjY1nNt1nHwxnH0zr0KYIgnqrHmkP1nznHTYPHDdPHTvrjbznsKzug7Y5HDLnWbsPWcLPjc4nj00Tv-b5H99PAc3mvD3njK-P1IWPhD0mLPV5Hb1nHRzrHbdPHbsrHbswbm0UAF15H00mynqnfKsUWYs0Z7VIjYs0Z7VT1Ys0ZGY5H00UyPxuMFEUHYsg1Kxn7ts0Aw9UMNBuNqsUA78pyw15HKxn7tsg100TA7Ygvu_myTqn0Kbmv-b5H00ugwGujYVnfK9TLKWm1Ys0ZNspy4Wm1Ys0Z7VuWYs0AuWIgfqn0KGTvP_5H00mywhUA7M5HD0UAuW5H00uAPWujY0IZF9uARqn0KBuA-b5HKjwjRzwHF7PYn3fYFKrH03f1uafbfswjcLf1n3nbns0AqW5HD0mMfqnfKEmgwL5H00ULfqn0KETMKY5H0WnanWnansc10Wna3snj0snj0WnaPDw-fWnanVc108nj0snj0sc1D8nj0snj0sc10WnansQW0snj0snansc10Wnansc10Wnans0AF9UhV9mvnqnansc10Wn0K3TLwd5HfkPWfdnHf10Z7xIWYsQW6zg108njKxna3sn7tsQW6zg108n1Ixn7tsQWnLg100mMPxTZFEuA-b5H00ThqGuhk9u1Ys0APv5fKGTdqWTADqn0KWTjYs0AN1IjYs0Z7MIvfqn0KETjDqn0KsTjfqn0KWThnqnWDznW6&us=newvui&xst=mWYsfYfdnbRzwHIjrDPafHbsrDnvfbFDnDfzPYn1rjFjn0715Hb4PWRznHnzPHnvrjf4nHbYnjFxnHfvnNts0gTqmvN_UaK1pyI8mykGUhT68pEQEoMu1x6KTHdWTLfKIHYYnHmYPHDYns7Y5HDLnWbsPWcLPjnKUgDqn0cs0BYKmv6quhPxTAnKUZRqn07WUWY4rj0knj03rNqbus7zIjYs0HndnHndrj01PHm&cegduid=PjDvPjRkPjn&solutionId=18733392&word=&ck=0.0.0.0.0.0.0.0&shh=www.baidu.com&sht=98010089_dg&wd=" \t "https://www.baidu.com/_blank) |
| Anti-β-Tubulin antibody | Rabbit | WB: 1:2500 | R20005 | Abmart |
| HRP-conjugated Goat Anti-Rabbit IgG | Goat | WB: 1:5,000  IHC:1:1000 | D110058 | Sangon |
| HRP-conjugated Goat Anti-Mouse IgG | Goat | WB: 1:5,000 | D110087 | Sangon |
| Dylight 549, Goat Anti-Rabbit IgG | Goat | IF:1:200 | A23320 | Abbkine |
| APC Anti-CD11b antibody | Rat | FC: 1:100 | 101212 | Biolegend |
| PerCP/Cy5.5 Anti-Ly6G antibody | Rat | FC: 1:100 | 108428 | Biolegend |
| Anti-CD47 antibody | Mouse | WB: 1:1500 | 66304-1-Ig | Proteintech |
| Anti-N-cadherin antibody | Rabbit | WB: 1:1500 | 22018-1-AP | Proteintech |


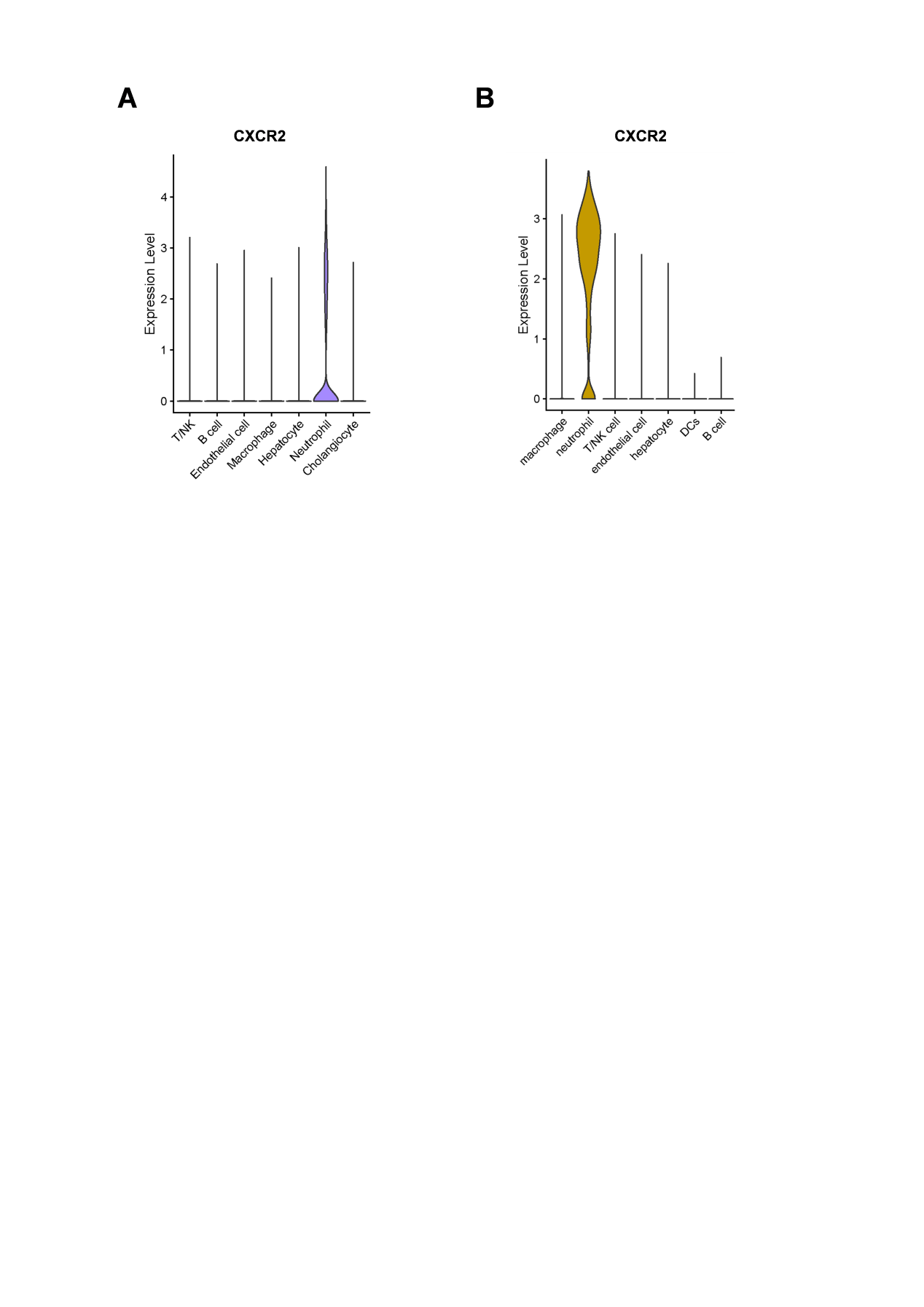


**Figure S1.** CXCR2 expression distribution across all cell types and quantitative analysis results. A：Human single cell sequencing dataset. B：Murine single cell sequencing dataset.


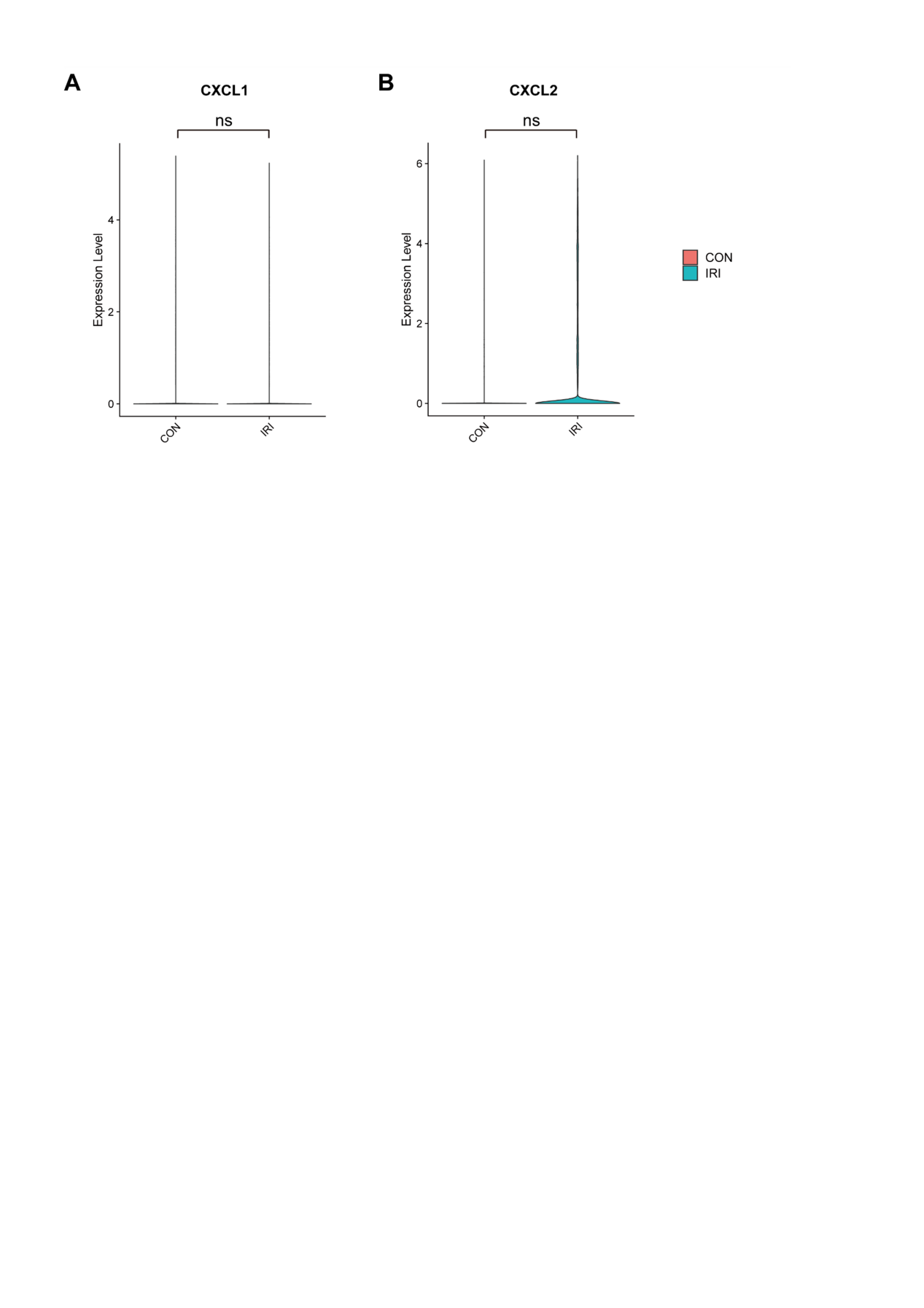


**Figure S2.** CXCL1 and CXCL2 expression distribution in lung from IRI and sham-operated groups ("ns" denotes no significance) GSE235367. A：CXCL1 expression. B：CXCL2 expression.


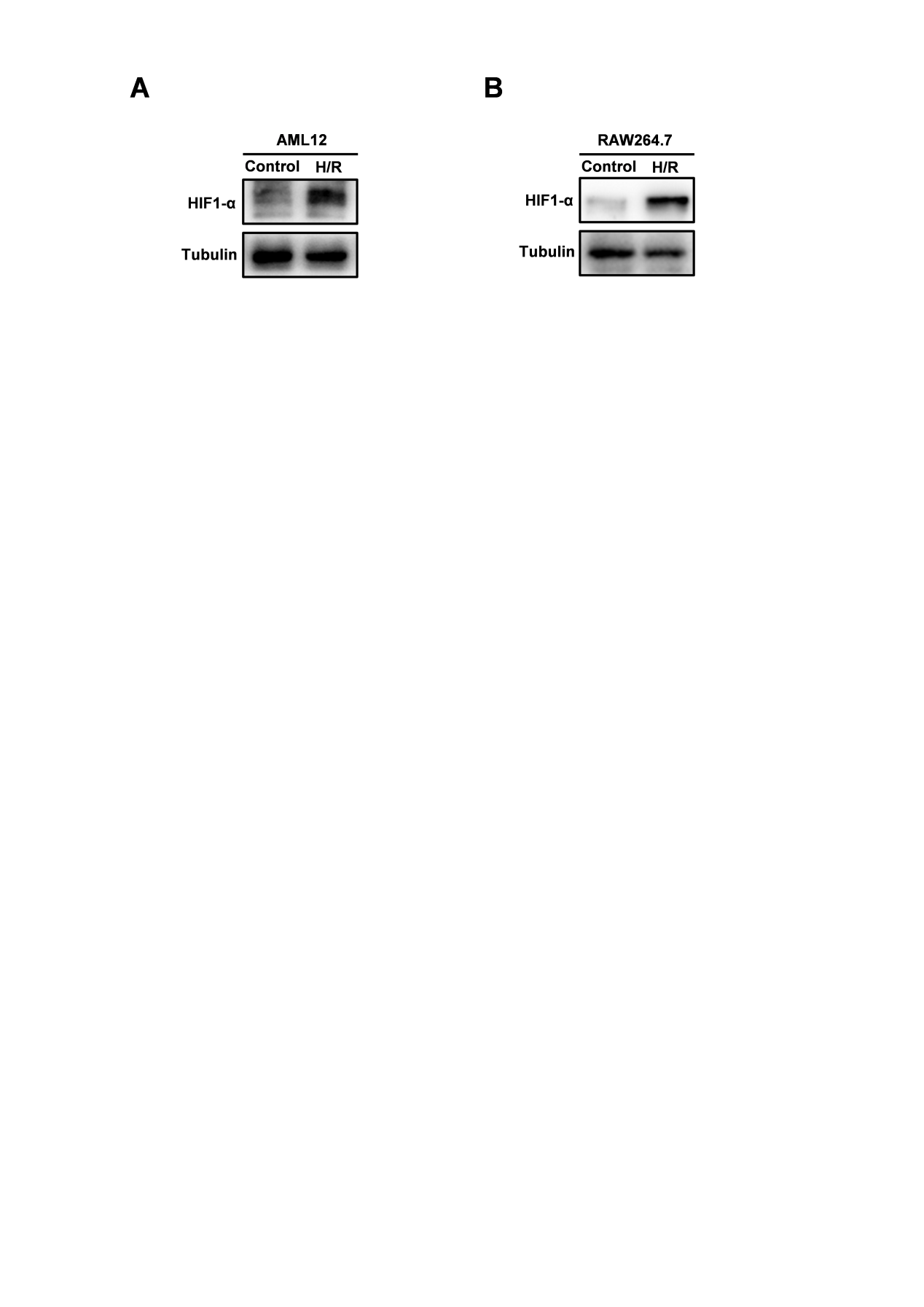


**Figure S3.** A-B: Western blot analysis of HIF1-α protein expression in RAW 264.7 and AML12 cells treated with hypoxia-reoxygenation. A：HIF1-α expression in AML12. B：HIF1-α expression in RAW 264.7.


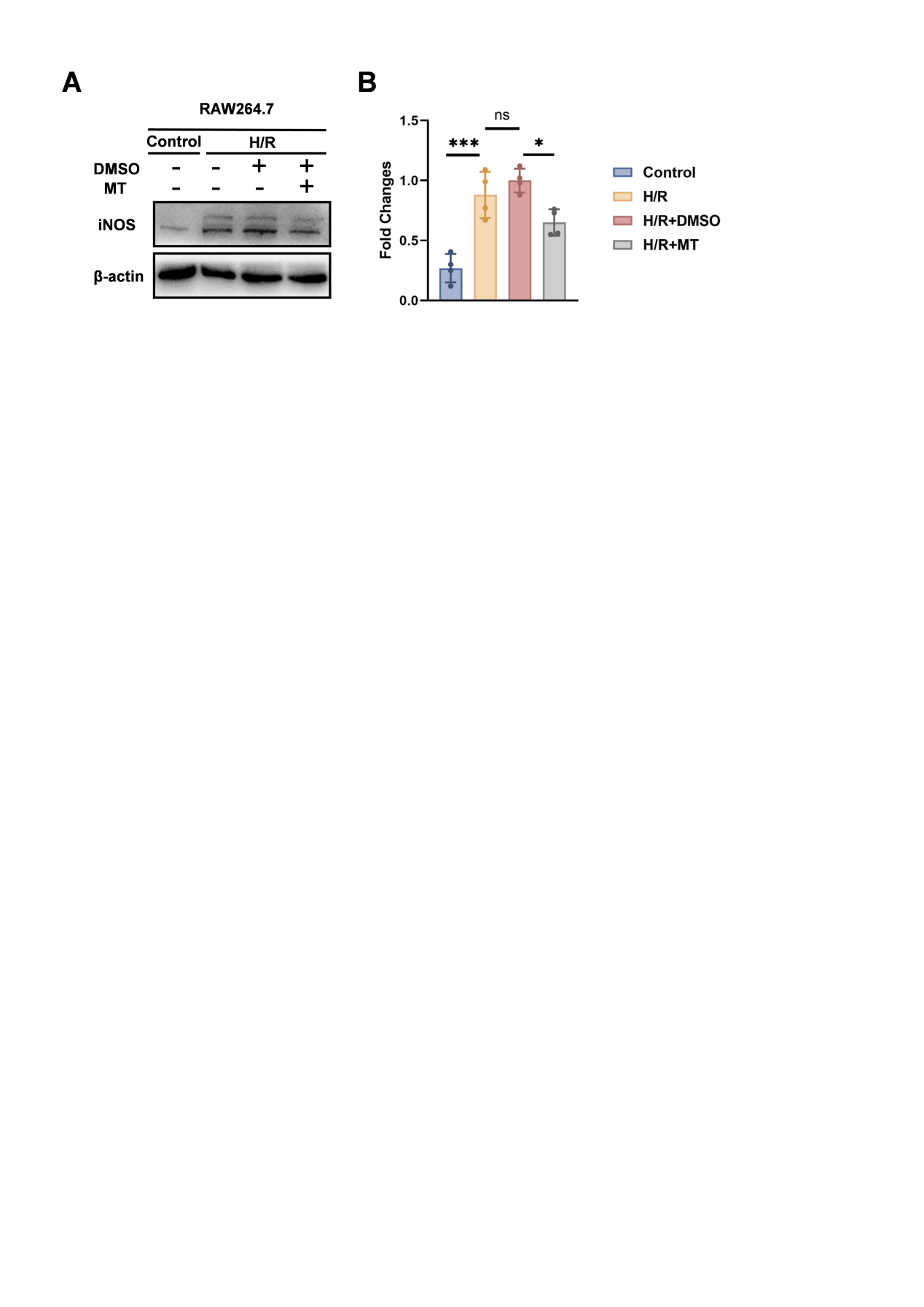


**Figure S4.** A-B: Western blot analysis of iNOS expression in RAW264.7 cells treated with hypoxia-reoxygenation and MT, showing their relative expression and quantification, with β-actin as a control (**p< 0.05, ***p< 0.001*).


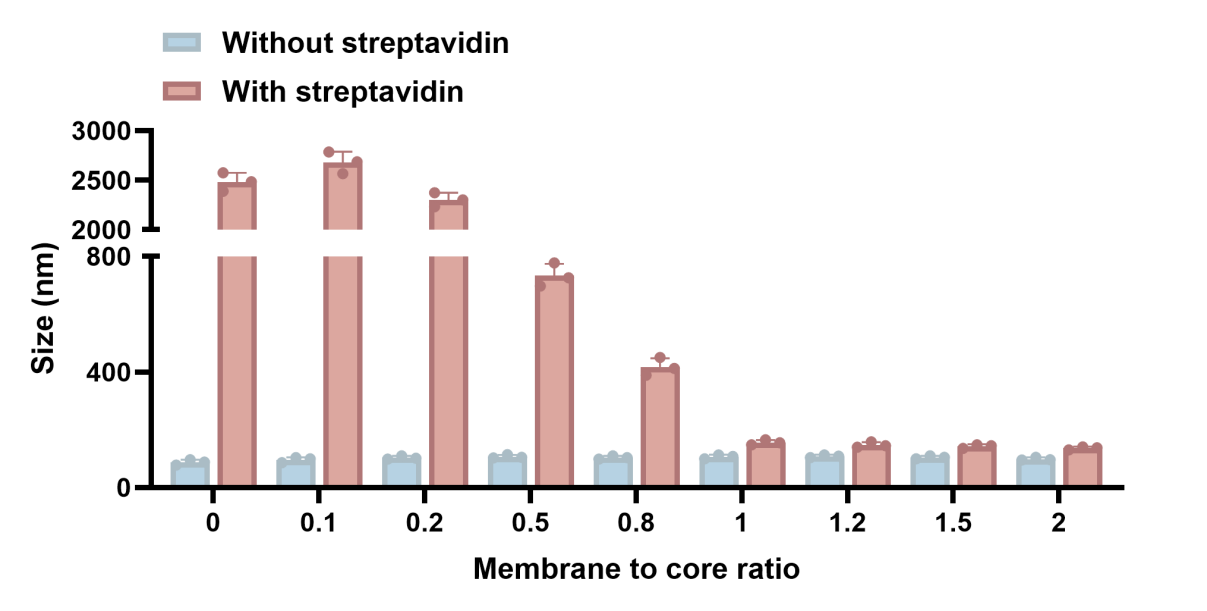


**Figure S5.** Size of biotinylated PLGA core when CXCR2-macrophage membranes were coated with different membrane/PLGA core weight ratios before and after incubation with streptavidin.


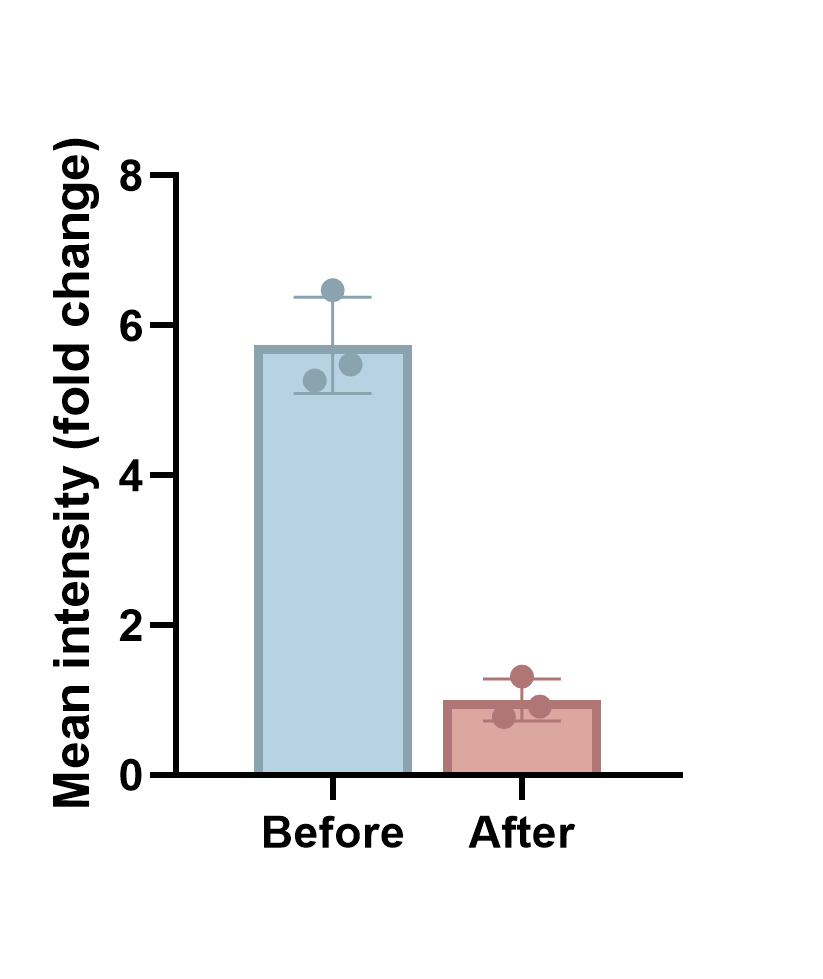


**Figure S6.** Membrane coating efficiency of CXCR2-MM@PLGA/MT nanoparticles. Fluorescence intensity in supernatant before and after centrifugation (15000 g, 10 min) of DilCXCR2-MM@PLGA/MT nanoparticles (1 mg/mL) (n = 3).


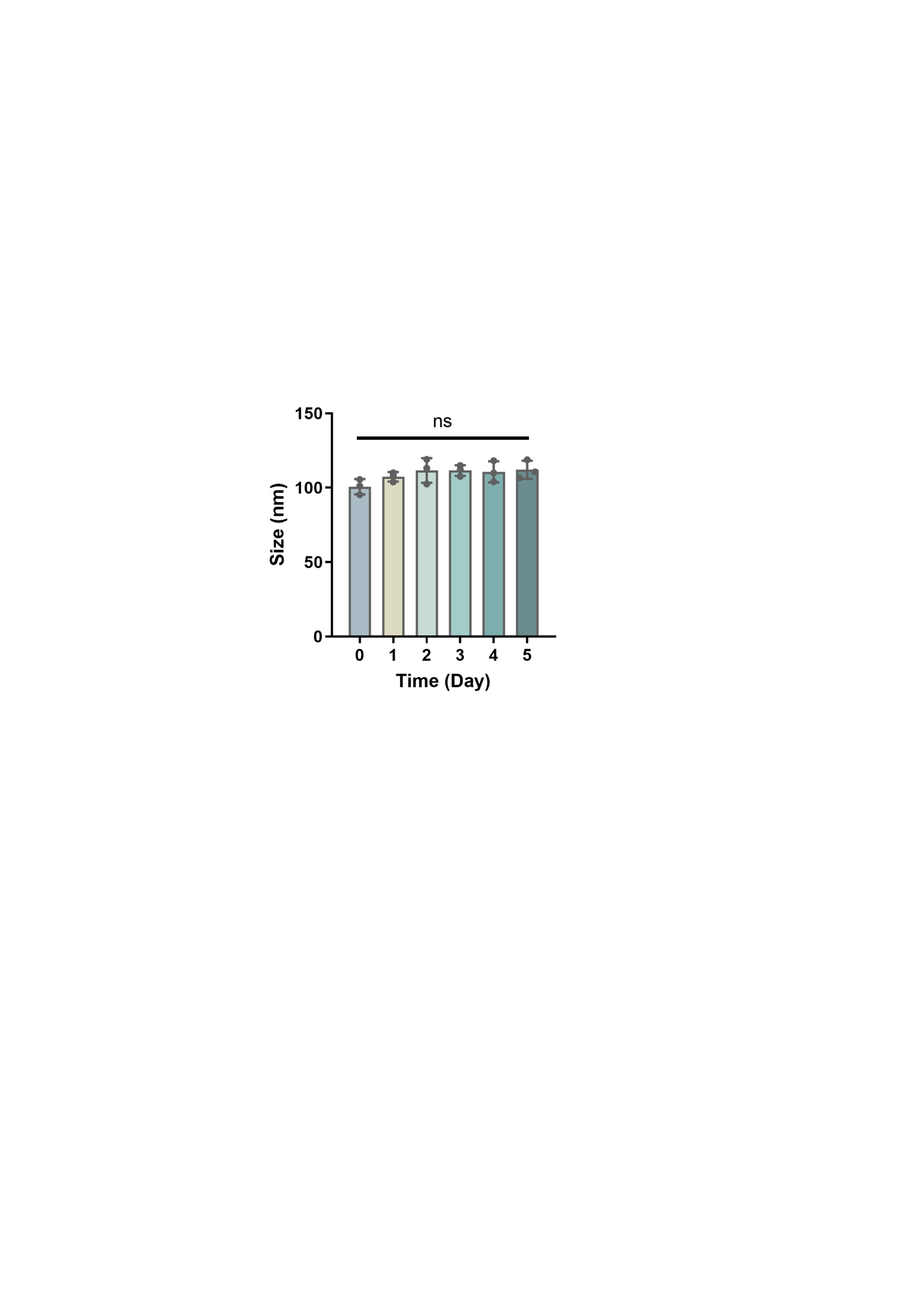


**Figure S7.** Mean diameter of CXCR2-MM@PLGA/MT after 0, 1, 2, 3, 4, and 5 days of storage in medium containing 10% fetal bovine serum at room temperature (*"ns" denotes no significance*), n=3.


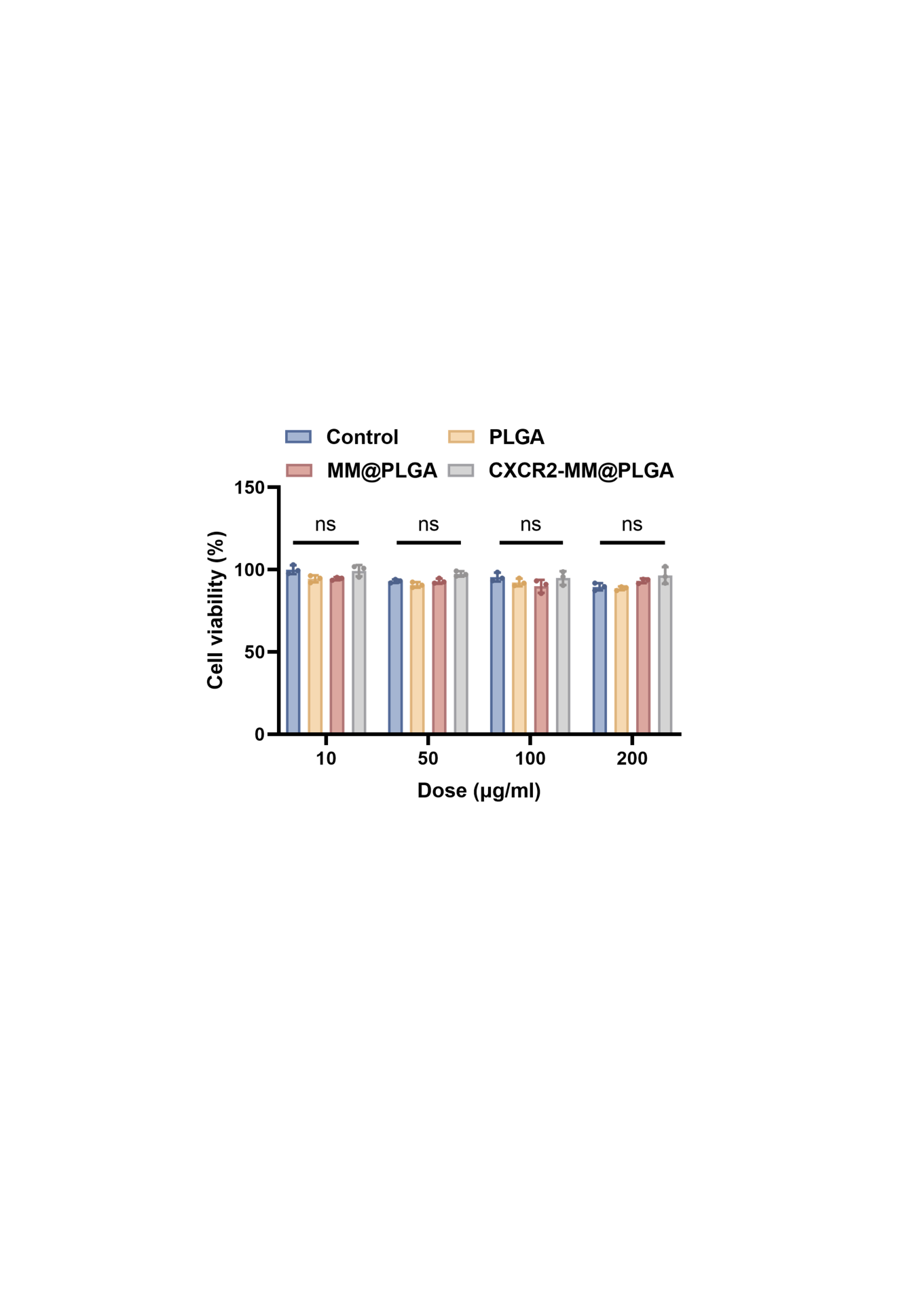


**Figure S8.** Cell survival of AML12 cells after 24 h of incubation with different concentrations of PLGA, MM@PLGA and CXCR2-MM@PLGA NPs was assayed by CCK-8(*"ns" denotes no significance*), n=3.


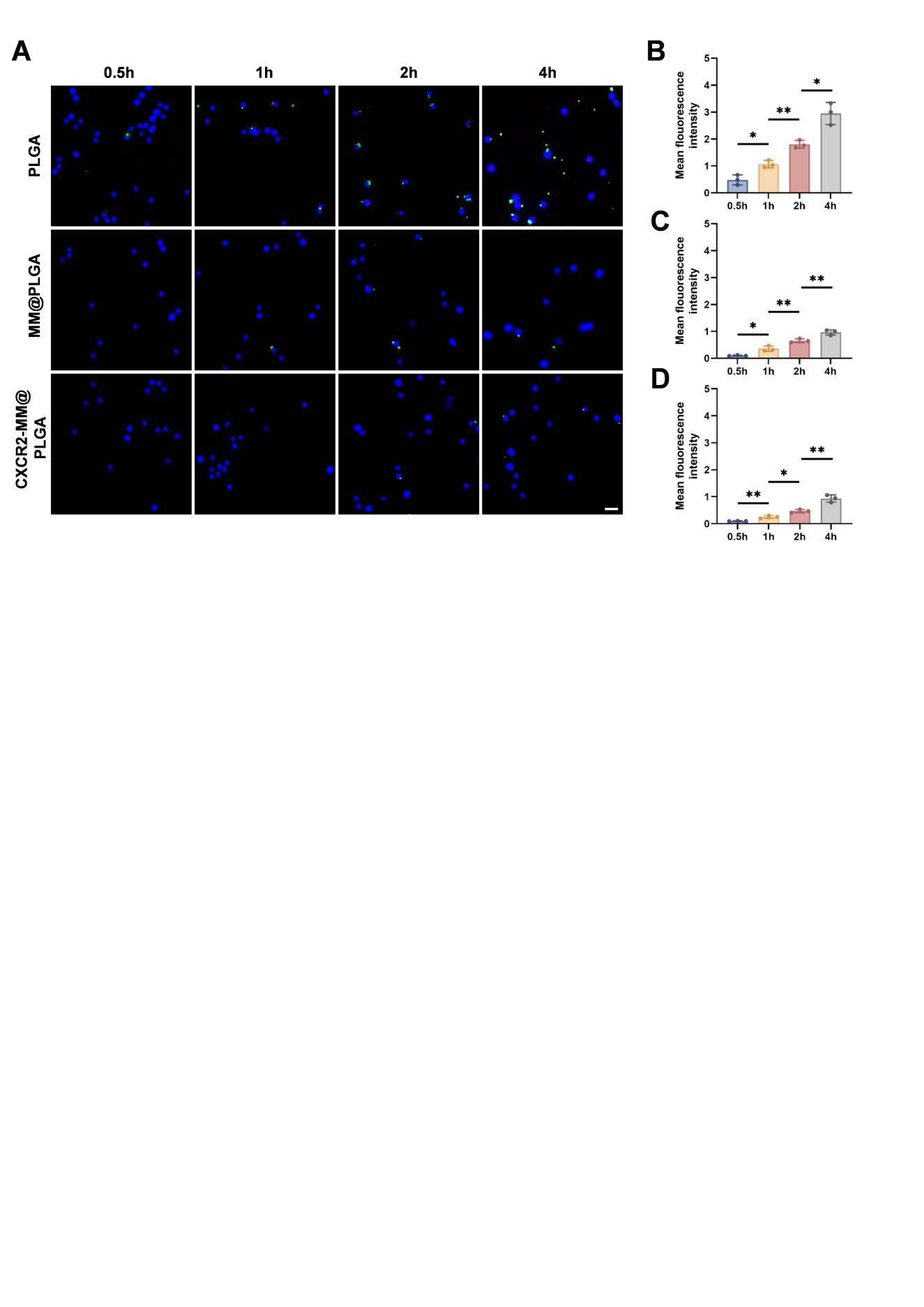


**Figure S9.** A-D: Representative fluorescence images showing phagocytosis of PLGA/coumarin6, MM@PLGA/coumarin6 and CXCR2-MM@PLGA/coumarin6 NPs by RAW 264.7 cells and including its quantitative analysis (**p< 0.05, **p< 0.01*) Scale bar = 20 nm, n=3.


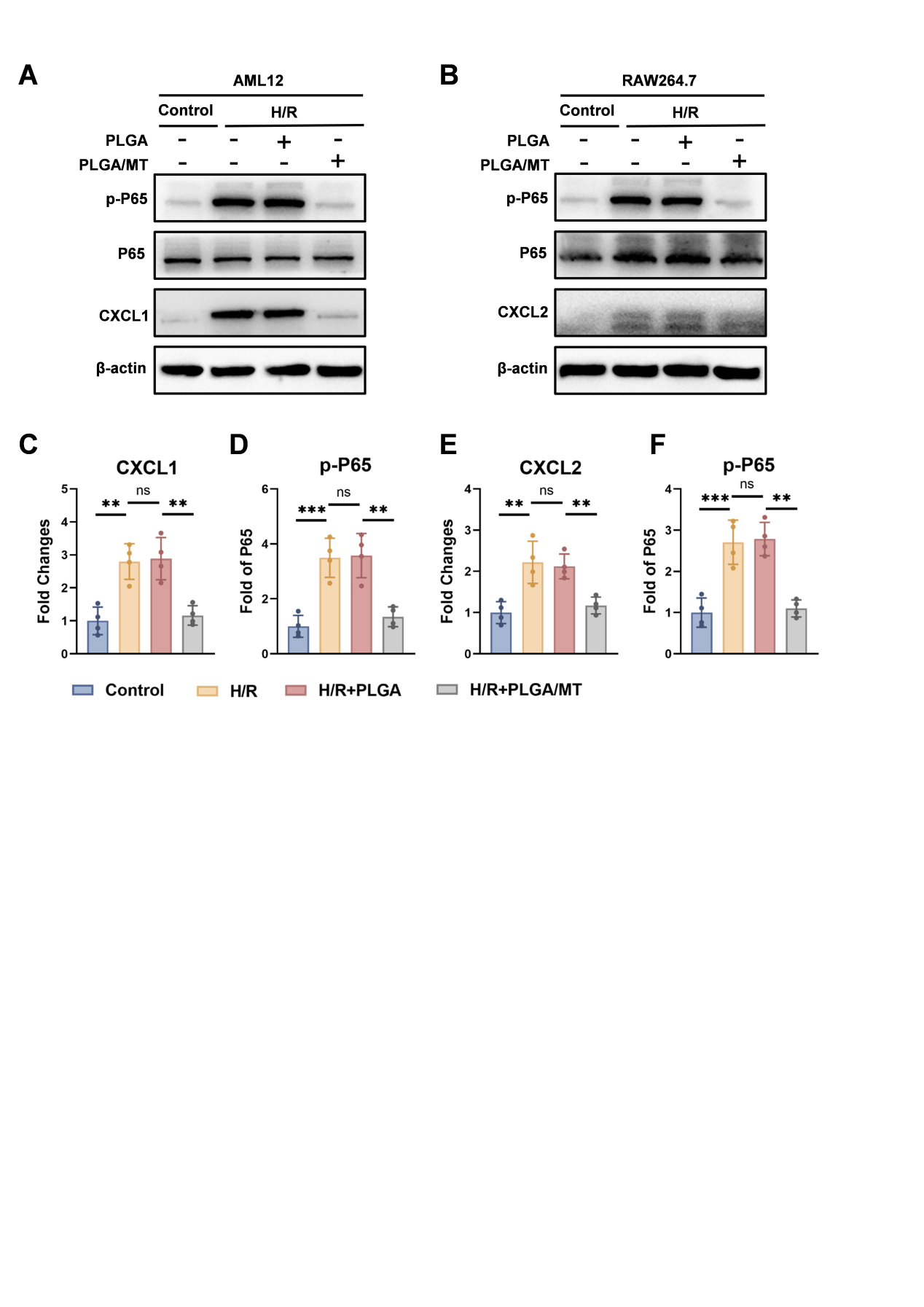


**Figure S10.** A-F: Western blot analysis of p-P65, P65, CXCL1, and CXCL2 expression in AML12 and RAW264.7 cells treated with hypoxia-reoxygenation and PLGA or PLGA/MT, showing their relative expression and quantification, with β-actin as a control (****p< 0.001, **p< 0.01*).


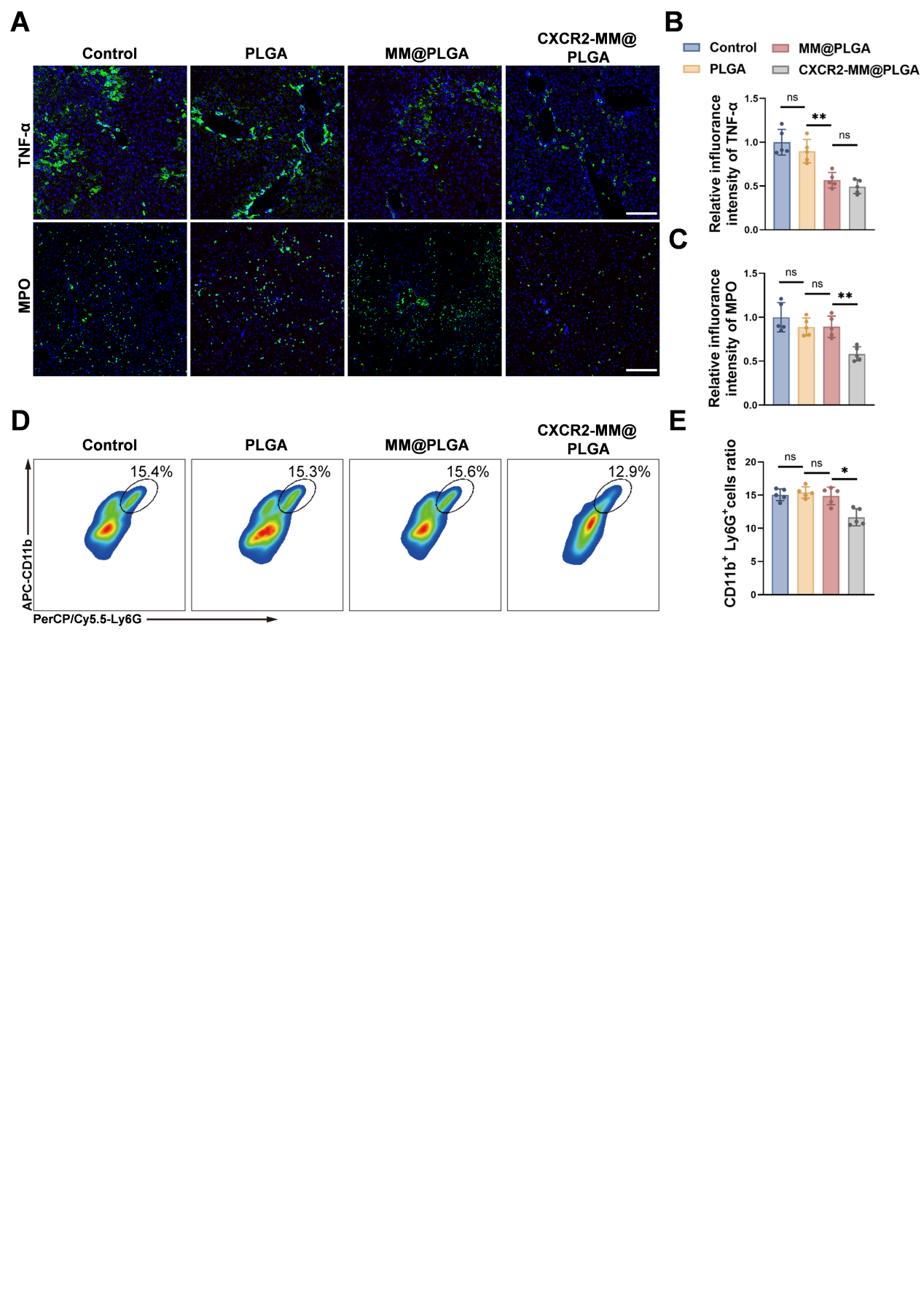


**Figure S11.** A: Immunofluorescence staining of TNF-α and MPO in mouse liver following IRI (n=5) Scale bar = 200 nm. B-C: Quantitative analysis of TNF-α+ and MPO+ cells from immunofluorescence images (*"ns" denotes no significance, **p< 0.01*). D: Flow cytometry analysis of neutrophil infiltration in mouse liver following IRI (n=5), with neutrophils labeled using APC-CD11b and PerCP/Cy5.5-Ly6G. E: Quantitative analysis of neutrophils infiltration based on flow cytometry results (*"ns" denotes no significance, *p<0.05*).
